# Strategyproof and fair matching mechanism for union of symmetric m-convex constraints

**DOI:** 10.1371/journal.pone.0289965

**Published:** 2024-02-08

**Authors:** Nathanaël Barrot, Kentaro Yahiro, Makoto Yokoo, Yuzhe Zhang

**Affiliations:** 1 EPF Graduate School of Engineering, Sceaux, France; 2 Kyushu University, Fukuoka, Japan; 3 Data61, CSIRO, Sydney, Australia; Gabriele d’Annunzio University of Chieti and Pescara Department of Economics: Universita degli Studi Gabriele d’Annunzio Chieti Pescara Dipartimento di Economia, ITALY

## Abstract

We identify a new class of distributional constraints defined as a union of symmetric M-convex sets, which can represent a wide range of real-life constraints in two-sided matching settings. Since M-convexity is not closed under union, a union of symmetric M-convex sets does not belong to this well-behaved class of constraints. Consequently, devising a fair and strategyproof mechanism to handle this new class is challenging. We present a novel mechanism for it called Quota Reduction Deferred Acceptance (QRDA), which repeatedly applies the standard Deferred Acceptance mechanism by sequentially reducing artificially introduced maximum quotas. We show that QRDA is fair and strategyproof when handling a union of symmetric M-convex sets, which extends previous results obtained for a subclass of the union of symmetric M-convex sets: ratio constraints. QRDA always yields a weakly better matching for students than a baseline mechanism called Artificial Cap Deferred Acceptance (ACDA). We also experimentally show that QRDA outperforms ACDA in terms of nonwastefulness.

## 1 Introduction

The objective of this paper is to identify a new class of distributional constraints, i.e., constraints denoted as a set of feasible allocation vectors, in two-sided matching, where a strategyproof and fair mechanism exists. In a two-sided matching problem, two types of agents (e.g., students/schools, hospitals/residents) are matched [1]. A standard matching market deals with maximum quotas (capacity limits). As the theory has been applied to diverse circumstances, mechanism designers have encountered various forms of distributional constraints. For instance, regional maximum quotas restrict the total number of students assigned to a set of schools [2]. Minimum quotas guarantee that each school accepts a certain number of students [3–7]. Furthermore, diversity constraints ensure a balanced mix of different student types within a school [8–12]. This topic has been receiving increased attention from AI researchers [13–17]. Throughout this paper, we focus on the context of a school-student allocation problem to enhance reader comprehension, although the results are applicable to general allocation problems.

The concept of stability is originally formulated by Gale and Shapley [18] for two-sided, one-to-one matching problems. In school-student matching problems, it is characterized as a combination of fairness and nonwastefulness. Fairness ensures that when a student, denoted as *s*, is not accepted by a school, denoted as *c*, despite considering it to be better than her assigned school, then *s* is ranked lower than any student accepted by *c*, based on *c*’s preference. Nonwastefulness is an efficiency notion that prohibits situations where a student can unilaterally move to a school she prefers without violating the underlying distributional constraints.

In standard matching markets with maximum quotas, the Deferred Acceptance (DA) mechanism finds a stable matching [18]. However, it is well-known that in the presence of distributional constraints, a stable matching may not exist. This presents a trade-off between fairness and efficiency for mechanism designers. Our approach investigates whether fairness can be satisfied under distributional constraints while maintaining as much efficiency as possible. As detailed below, a baseline mechanism called Artificial Cap Deferred Acceptance (ACDA), which utilizes predetermined artificial maximum quotas, is strategyproof and fair. However, it does not perform well in terms of nonwastefulness, since it compromises much of the original distributional constraints’ flexibility.

We restrict our attention to strategyproof mechanisms, which are mechanisms ensuring that no student has an incentive to misreport her preference over schools. In theory, if we are interested in a property achieved in dominant-strategies, we can restrict our attention to strategyproof mechanisms without losing generality as the well-known revelation principle [19]. This principle states that if a particular property is satisfied in a dominant strategy equilibrium using a certain mechanism, it can also be attained by a strategyproof mechanism. A strategyproof mechanism is also useful in practice since a student does not need to speculate about the actions of other students to obtain a good outcome; she only needs to truthfully report her preference.

Existing works have shown that if constraints belong to a well-behaved class in discrete convex analysis, called an M-convex set, then the Generalized Deferred Acceptance mechanism is strategyproof, fair, and preserves the flexibility of the original distributional constraints [20]. The character “M” within the term “M-convexity” symbolizes the concept of matroid, a concept used in Kojima et al. [20] to define the condition under which the Generalized Deferred Acceptance mechanism satisfies desirable properties. As described in our model, their condition can be translated to an M-convex set. In consequence, the pursuit of an alternative class beyond M-convex sets for which we can develop a non-trivial strategyproof and fair mechanism, is theoretically interesting and challenging.

In this paper, we introduce a new class of constraints denoted as *a union of symmetric M-convex sets*. Note that M-convexity is not closed under union. For example, since a set with just one vector is an M-convex set, any set of vectors (with an identical *L*_1_ norm) can be represented by a union of M-convex sets. Therefore, a union of symmetric M-convex sets is not M-convex. However, it forms a captivating class of distributional constraints encompassing a diverse range of real-life constraints relevant to two-sided matching. An exemplary instance of such constraints is the ratio constraints [21], which specify the acceptable minimum ratio between the least and most popular schools (Definition 15).

**Fig 1:**
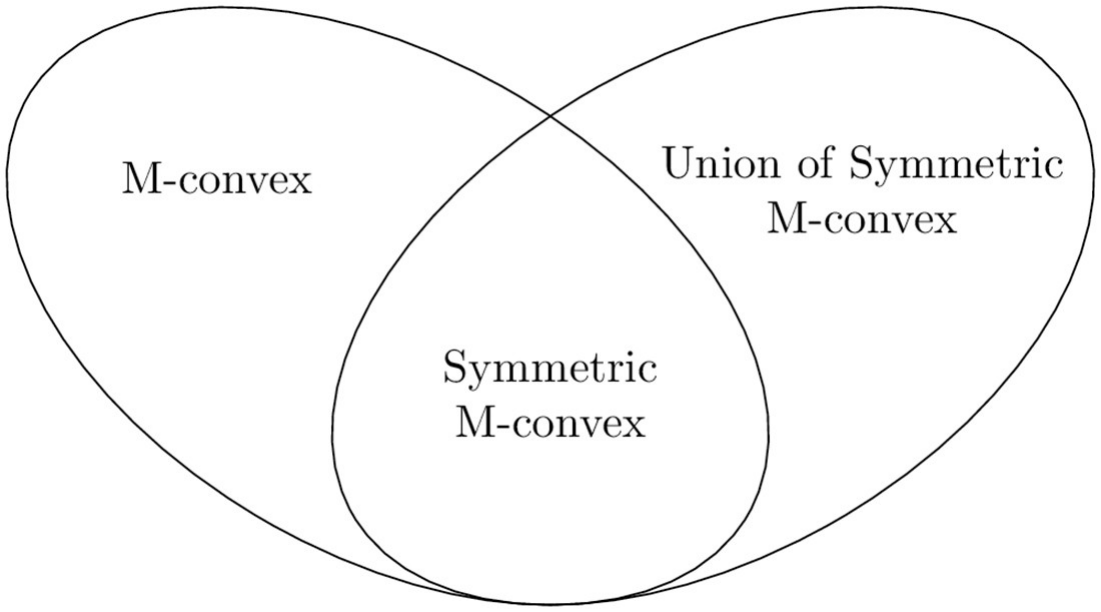
Inclusion of notions in discrete convex analysis.

We develop a strategyproof and fair mechanism called Quota Reduction Deferred Acceptance (QRDA), which repeatedly applies DA by sequentially reducing artificially introduced maximum quotas. QRDA generalizes another mechanism, also referred to as QRDA, which we specifically devised to address ratio constraints [21]. The class of distributional constraints we study is a strict generalization of ratio constraints. Fig 1 illustrates the inclusion relationship among the concepts explored in this paper. Since the set of unions of symmetric M-convex sets is symmetric, its intersection with the set of M-convex sets corresponds to the set of symmetric M-convex sets.

To the best of our knowledge, we are the first to identify a general class of constraints beyond M-convex sets, wherein a non-trivial, strategyproof, and fair mechanism exists. Through the development of innovative proof techniques, we show that QRDA is fair and strategyproof under the union of symmetric M-convex constraints. In terms of student welfare, we establish the weak Pareto optimality of QRDA and prove that no strategyproof mechanism can dominate QRDA. We also theoretically show that QRDA outperforms another fair mechanism, ACDA, and extend these results for an asymmetric generalization of the union of symmetric constraints. In terms of nonwastefulness, we conduct experiments that reveal QRDA’s superiority over ACDA. Additionally, we expand these experiments to compare QRDA with an additional mechanism, Extended Seat Deferred Acceptance (ESDA) [4], yielding similar conclusions.

This paper builds upon our conference paper [22]. The following are the main differences: full proofs (Lemmas 3 and 4, and Theorem 2), an extended study of QRDA’s axiomatic properties (Theorem 3, 4, 5, and 6), a generalized asymmetric model (Section 5), and an extended comparison with existing mechanisms for distributional constraints (theoretically with Theorem 11 and experimentally with extended simulations).

### 1.1 Related work

Our mechanism achieves strategyproofness and fairness through a sequential reduction of artificial quotas combined with the application of DA. The idea of sequentially reducing maximum quotas is also used by Fragiadakis and Troyan [23], albeit for a different objective. In their model, students are categorized into different types, and the objective is to fulfill type-specific minimum and maximum quotas.

Several papers have investigated strategyproofness in matching models with quotas constraints, although they consider a setting distinct from ours [24–27]. These papers address a student-course setting where only students express preferences over courses, and both students and courses have quotas or capacities. In contrast, our setting involves preferences from both students and schools, with only schools having constraints. A study closer to our setting is conducted by Kamada and Kojima [28], who investigate student-school matchings and characterize the constraints referred to as *general upper bounds*, ensuring the existence of a student-optimal and fair matching. However, they consider constraints imposed on individual schools whereas our constraints are symmetric over the set of schools. Moreover, they allow constraints to depend on the identity of students, whereas our constraints are solely based on the number of students.

Two streams of work exist on matching with distributional constraints. One stream scrutinizes constraints that arise from real-life applications, such as regional maximum quotas [2], individual/regional minimum quotas [4, 5], affirmative actions [9, 11], and ratio constraints [21]. The other stream mathematically investigates an abstract and general class of constraints, exemplified by those represented by a substitute choice function [29], an M-convex set [20], and a general upper bound [28]. The results derived from the first stream are more accessible due to their practical grounding and direct applicability to real-life scenarios. In contrast, the results obtained from the second stream are rigorously organized mathematically and have broader applicability.

Our paper belongs to the second stream. In particular, it builds upon our previous work [21], which belongs to the first stream focusing on ratio constraints (Definition 15). These constraints specify the acceptable minimum ratio between the least and most popular schools (Definition 15). The class of distributional constraints we study is a strict generalization of ratio constraints, and it is defined based on a well-known concept in discrete convex analysis: M-convex sets. Although our QRDA mechanism uses a similar idea as a mechanism for ratio constraints, such as the sequential reduction of quotas, the proof techniques for our main results, notably strategyproofness with Theorem 2, differ significantly. This distinction arises due to the reliance of proof techniques for ratio constraints on specific constraint structures, rendering them inapplicable to our setting. Consequently, these proof techniques need to undergo adaptations to suit our setting.

### 1.2 Outline

Section 2 introduces the model used to study the proposed matching problem and defines the desirable properties. In section 3, the family of M-convex sets is introduced, and we go beyond this family by defying the union of symmetric M-convex constraints. Subsequently, the main matching mechanism, QRDA, is described in Section 4, accompanied by proofs of its properties. Additionally, QRDA is theoretically compared with a baseline mechanism, ACDA. In Section 5, we generalize the symmetric constraints to the asymmetric space, demonstrating QRDA’s adaptability. Empirical comparisons of several matching mechanisms are conducted in Section 6. Finally, this paper concludes in Section 7.

All technical notations used in this paper are listed in Table 1.

**Table 1:** Notations used in this paper.

Notation	Term
*S* = {*s*_1_, …, *s*_*n*_}	Set of *n* students.
*C* = {*c*_1_, …, *c*_*m*_}	Set of *m* schools.
${≻}_{S}=\left({≻}_{s_{1}},\ldots ,{≻}_{s_{n}}\right)$	The profile of student preferences.
${≻}_{C}=\left({≻}_{c_{1}},\ldots ,{≻}_{c_{m}}\right)$	The profile of school preferences.
$V\subseteq N_{0}^{m}$	The set of school-feasible vectors, denoting the distributional constraints.
*X* = *S* × *C*	The set of all possible contracts.
For $\dot{X}\subseteq X$, ${\dot{X}}_{s}$	The set of contracts related to *s*.
For $\dot{X}\subseteq X$, ${\dot{X}}_{c}$	The set of contracts related to *c*.
$\zeta (\dot{X})$	The allocation vector of $\dot{X}$.
$\varphi \left({≻}_{S}\right)=\dot{X}$	Matching mechanism *φ* takes ≻_*S*_ and outputs matching $\dot{X}$.
(≻_*s*_, ≻_*S*\{*s*}_)	All students take preference ≻_*S*_ except for *s*, who takes ≻_*s*_.
*U*(≻_*s*_, *c*) = {*c*′ ∈ *C*∣*c*′≻_*s*_ *c*}	The strict upper contour of *c*, i.e., all schools that *s* prefers over school *c*.
*χ*_*i*_	*m*-dimensional unit vector where the *i*-th element is 1.
*α*	A parameter used in Ratio constraint.
*β*	A parameter used in Difference constraint.
*p*_*c*_/*q*_*c*_	The minimum/maximum quota of school *c*.
*σ*	A sequence of schools based on the round-robin order *c*_1_, *c*_2_, …, *c*_*m*_.
*ν* ^*max*^	An upper-bound for the value of all elements in all feasible vectors in V.
$\nu_{c}^{max}$	An upper-bound for the value of all elements in $\nu_{c}\in V$.
$q_{c}^{k}$	The maximum quota of school *c* in the *k*-th stage of QRDA.
${\dot{X}}^{k}$	The matching obtained in the *k*-th stage of QRDA.
$C_{s}$	A scenario of student *s*.
$R(C_{s})$	A rejection chain of a scenario of student *s*.
*η*	An operation that transforms a vector.
*θ*	The spread parameter of the Mallows model.
*ω*(≻_*s*_, ≻_*s*′_)	The Kendall tau distance between preferences ≻_*s*_ and ≻_*s*′_.

## 2 Model

A student-school matching market with distributional constraints is defined by a tuple $\left(S,C,{≻}_{S},{≻}_{C},V\right)$. For brevity, given a positive integer *i*, we use [*i*] = {1, 2, …, *i*}.

- *S* = {*s*_1_, …, *s*_*n*_} is a finite set of *n* students.
- *C* = {*c*_1_, …, *c*_*m*_} is a finite set of *m* schools.
-
${≻}_{S}=\left({≻}_{s_{1}},\ldots ,{≻}_{s_{n}}\right)$ is the profile of the student preferences, where each ≻_*s*_ is a strict and complete preference order over *C*. For example, if *s* strictly prefers *c* over *c*′, it is denoted by *c* ≻_*s*_ *c*′. Moreover, we denote by *c* ⪰ _*s*_*c*′ if either *c* ≻_*s*_ *c*′ or *c* = *c*′.
-
${≻}_{C}=\left({≻}_{c_{1}},\ldots ,{≻}_{c_{m}}\right)$ is the profile of the school preferences, where each ≻_*c*_ is a strict and complete preference over *S*. For example, if *c* strictly prefers *s* over *s*′, it is denoted by *s* ≻_*c*_ *s*′. Similarly, we denote by *s* ⪰_*c*_ *s*′ if either *s* ≻_*c*_ *s*′ or *s* = *s*′.
-
$V\subseteq N_{0}^{m}$ is a set of school-feasible vectors that reflects distributional constraints. For each ν∈V, we assume ∑_*i*∈[*m*]_*ν*_*i*_ = *n* holds.

Set *X* = *S* × *C* is a finite set of all possible contracts. Contract (*s*, *c*) ∈ *X* means that student *s* is matched to school *c*. For $\dot{X}\subseteq X$, ${\dot{X}}_{s}$ denotes $\left\{\left(s,c\right)\in \dot{X}|c\in C\right\}$, and ${\dot{X}}_{c}$ denotes $\left\{\left(s,c\right)\in \dot{X}|s\in S\right\}$. In other words, ${\dot{X}}_{s}$ (respectively, ${\dot{X}}_{c}$) denotes all contracts in $\dot{X}$ related to *s* (respectively, *c*). For $\dot{X}$, let $\zeta (\dot{X})$ denote the *m*-element vector $\left(|{\dot{X}}_{c_{1}}|,|{\dot{X}}_{c_{2}}|,\ldots ,|{\dot{X}}_{c_{m}}|\right)$. We call $\zeta (\dot{X})$ the *allocation vector* of matching $\dot{X}$.

**Definition 1** (Feasibility). *For* $\dot{X}\subseteq X$, $\dot{X}$ is student-feasible if $|{\dot{X}}_{s}|=1$ *for all s* ∈ *S*. *A set of contracts is called a matching if it is student-feasible. Set* $\dot{X}$ *is school-feasible if* $\zeta (\dot{X})\in V$. *A set of contracts is feasible if it is both student-/school-feasible*.

In this market, we assume that all schools are acceptable to all students and vice versa. This strong assumption is necessary to guarantee the existence of a feasible matching, and it is commonly used in previous works [4, 5, 12, 30].

With a slight abuse of notation, for two matchings, $\dot{X}$ and $\ddot{X}$, we write ${\dot{X}}_{s}{≻}_{s}\;{\ddot{X}}_{s}$ if ${\dot{X}}_{s}=\left\{\left(s,c^{\prime }\right)\right\}$, ${\ddot{X}}_{s}=\left\{\left(s,c^{\prime \prime }\right)\right\}$, and *c*′ ≻_*s*_ *c*′′ (i.e., if student *s* prefers the school she obtained in $\dot{X}$ to the one in $\ddot{X}$). Furthermore, if either ${\dot{X}}_{s}{≻}_{s}\;{\ddot{X}}_{s}$ or ${\dot{X}}_{s}={\ddot{X}}_{s}$, we denote ${\dot{X}}_{s}{≽}_{s}\;{\ddot{X}}_{s}$, which reads as student *s* *weakly* prefers ${\dot{X}}_{s}$ over ${\ddot{X}}_{s}$.

Mechanism *φ* is a function that takes a profile of student preferences ≻_*S*_ as input and returns a set of contracts. Given that $\varphi \left({≻}_{S}\right)=\dot{X}$, let *φ*_*s*_(≻_*S*_) denote ${\dot{X}}_{s}$. Let ≻_*S*\{*s*}_ denote the profile of the preferences where all students’ preferences are ≻_*S*_ except *s*, whose preference is ≻_*s*_. Note that we assume that the profile of school preferences is publicly known and focus on strategyproofness (Definition 2) for students (the proposing side). Thus, we do not specify the profile of school preferences as an input of a mechanism.

In this paper, we study three desirable mechanism properties in the context of matching: *strategyproofness*, *fairness*, and *nonwastefulness*. Strategyproofness guarantees that students always have an incentive to truthfully report their preferences. A mechanism satisfies strategyproofness if no profile exists where a student can benefit from individually misreporting.

**Definition 2** (Strategyproofness). *Mechanism φ is strategyproof if for all preference profile* ≻_*S*_, *no student s* ∈ *S and* ${≻}_{s}^{\prime }$ *(a possible misreport of s*’*s preference) exist such that* $\varphi_{s}\left(\left({≻}_{s}^{\prime },{≻}_{S\backslash \{s\}}\right)\right){≻}_{s}\;\varphi_{s}\left(\left({≻}_{s},{≻}_{S\backslash \{s\}}\right)\right)$.

A stronger requirement than strategyproofness is group strategyproofness, which ensures that no group of students can benefit from collectively misreporting.

**Definition 3** (Weak/Strong Group Strategyproofness). *Mechanism φ is weakly group strategyproof if for all preference profile* ≻_*S*_, *no group of students S*′ ⊆ *S and* ${≻}_{S^{\prime }}^{\prime }$ *(a possible collusive misreport by S*′) *exist such that for all s* ∈ *S*′, $\varphi_{s}\left(\left({≻}_{S^{\prime }}^{\prime },{≻}_{S\backslash S^{\prime }}\right)\right){≻}_{s}\varphi_{s}\left(\left({≻}_{S^{\prime }},{≻}_{S\backslash S^{\prime }}\right)\right)$. *Mechanism φ is strongly group strategyproof if for all preference profile* ≻_*S*_, *no group of students S*′ ⊆ *S and* ${≻}_{S^{\prime }}^{\prime }$ *(a possible collusive misreport by S*′) *exist such that for all s* ∈ *S*′, $\varphi_{s}\left(\left({≻}_{S^{\prime }}^{\prime },{≻}_{S\backslash S^{\prime }}\right)\right){⪰}_{s}\;\varphi_{s}\left(\left({≻}_{S^{\prime }},{≻}_{S\backslash S^{\prime }}\right)\right)$ *and for some s* ∈ *S*′, $\varphi_{s}\left(\left({≻}_{S^{\prime }}^{\prime },{≻}_{S\backslash S^{\prime }}\right)\right){≻}_{s}\varphi_{s}\left(\left({≻}_{S^{\prime }},{≻}_{S\backslash S^{\prime }}\right)\right)$.

Fairness is defined through the notion of *justified envy* and it has been studied in constrained matching models [31, 32]. Given a specific matching, student *s* has justified envy toward student *s*′, if *s*′ is assigned to school *c*′ which *s* prefers to *s*’s current school, despite the fact that *c*′ prefers *s* over *s*′.

**Definition 4** (Fairness). *In matching* $\dot{X}$, *where* $\left(s,c\right)\in \dot{X}$, *student s has justified envy toward another student s*′ *if for some c*′ ∈ *C*, $\left(s^{\prime },c^{\prime }\right)\in \dot{X}$, (*s*, *c*′) ≻_*s*_ (*s*, *c*), *and* (*s*, *c*′) ≻_*c*′_ (*s*′, *c*′) *hold. Matching* $\dot{X}$ *is fair if no student has justified envy in* $\dot{X}$. *A mechanism is fair if it always produces a fair matching*.

Nonwastefulness is concerned with the efficiency of a mechanism and guarantees that no student *claims* an empty seat. Given a specific matching, student *s* claims an empty seat in *c*′ if she prefers *c*′ to her current school *c* and if the matching obtained by moving her from *c* to *c*′ remains school-feasible.

**Definition 5** (Nonwastefulness). *In matching* $\dot{X}$ *where* $\left(s,c\right)\in \dot{X}$, *student s claims an empty seat in c*′ *if* (*s*, *c*′) ≻_*s*_ (*s*, *c*) *and* $\left(\dot{X}\backslash \left\{\left(s,c\right)\right\}\right)\cup \left\{\left(s,c^{\prime }\right)\right\}$ *is school-feasible. Matching* $\dot{X}$ *is nonwasteful if no student claims an empty seat in* $\dot{X}$. *A mechanism is nonwasteful if it always produces a nonwasteful matching*.

In standard matching terminology, fairness and nonwastefulness are combined to form a notion called *stability* [5, 23, 30]. However, these two properties are generally incompatible under distributional constraints. In particular, they are incompatible in our setting, as demonstrated in Section 3.2.2. Thus, in this paper, we decompose the stability notion into fairness and nonwastefulness and focus on *finding a fair outcome while reducing wastefulness* as much as possible. Dividing stability into fairness and nonwastefulness is commonly used when dealing with distributional constraints [2, 4, 20, 30].

In addition to strategyproofness, fairness, and nonwastefulness, another well-studied efficiency notion is *Pareto optimality*, which requires that no other matching exists where all students are weakly better off.

**Definition 6** (Pareto Optimality and Domination). *Matching* $\dot{X}$ *strongly dominates matching* $\ddot{X}$ if ${\dot{X}}_{s}{≻}_{s}{\ddot{X}}_{s}$ *holds for every s* ∈ *S*. *Matching* $\dot{X}$ *weakly dominates matching* $\ddot{X}$ if ${\dot{X}}_{s}{⪰}_{s}{\ddot{X}}_{s}$ *holds for every s* ∈ *S*, *and s* ∈ *S exists such that* ${\dot{X}}_{s}{≻}_{s}{\ddot{X}}_{s}$ *holds. Mechanism φ dominates mechanism ψ if for each preference profile of students* ≻_*S*_, *either φ*(≻_*S*_) *weakly dominates ψ*(≻_*S*_) *or φ*(≻_*S*_) = *ψ*(≻_*S*_) *holds, and profile* ≻_*S*_ *exists such that φ*(≻_*S*_) *weakly dominates ψ*(≻_*S*_). *Matching* $\dot{X}$ *is weakly (respectively, strongly) Pareto optimal for students if no matching* $\ddot{X}$ *exists that strongly (respectively, weakly) dominates* $\dot{X}$. *Furthermore, a mechanism is weakly (respectively, strongly) Pareto optimal if it always produces a weakly (respectively, strongly) Pareto optimal matching for students*.

Finally, we define two properties, *weak non-bossiness* and *weak Maskin monotonicity*, which are related to weak group strategyproofness. First, we present some definitions to describe these properties.

The *strict upper contour* set of school *c* at preference ≻_*s*_, denoted as *U*(≻_*s*_, *c*), is the set of schools that student *s* strictly prefers to school *c*:

$$\begin{array}{c} U\left({≻}_{s},c\right)=\left\{c^{\prime }\in C|c^{\prime }\;{≻}_{s}\;c\right\}. \end{array}$$

Preference ${≻}_{s}^{\prime }$ is a *monotonic transformation* of preference ≻_*s*_ at school *c* (or equivalently at contract (*s*, *c*)) if $U\left({≻}_{s}^{\prime },c\right)\subseteq U\left({≻}_{s},c\right)$. In other words, the set of schools preferred to *c* in ${≻}_{s}^{\prime }$ is a subset of the schools preferred to *c* in ≻_*s*_. Preference ${≻}_{s}^{\prime }$ is an *upper-contour-set preserving transformation* of ≻_*s*_ at school *c* (or equivalently at contract (*s*, *c*)) if $U\left({≻}_{s}^{\prime },c\right)=U\left({≻}_{s},c\right)$. Informally, the set of schools preferred to *c* in ${≻}_{s}^{\prime }$ is exactly the set of schools preferred to *c* in ≻_*s*_.

These notions naturally extend to the preference profiles. Profile ${≻}_{S}^{\prime }$ is a monotonic transformation (respectively, upper-contour-set preserving transformation) of profile ≻_*S*_ at matching $\dot{X}$ if for each student *s*, preference ${≻}_{s}^{\prime }$ is a monotonic transformation (respectively, upper-contour-set preserving transformation) of preference ≻_*s*_ at contract ${\dot{X}}_{s}$.

**Definition 7** (Weak Non-bossiness). *Mechanism φ is weakly non-bossy if for any preference profile* ≻_*S*_, *student s and preference* ${≻}_{s}^{\prime }$ *such that* ${≻}_{s}^{\prime }$ *is an upper-contour-set preserving transformation of* ≻_*s*_ at *φ*_*s*_(≻_*S*_), *the following holds*:

$$\begin{array}{c} \left[\varphi_{s}\left({≻}_{s}^{\prime },{≻}_{S\backslash \{s\}}\right)=\varphi_{s}\left({≻}_{S}\right)\right]\Rightarrow \left[\varphi \left({≻}_{s}^{\prime },{≻}_{S\backslash \{s\}}\right)=\varphi \left({≻}_{S}\right)\right]. \end{array}$$

This is a weaker version of a property called non-bossiness, which was first introduced by Satterthwaite and Sonnenschein [33]. Non-bossiness demands that a student cannot alter the assignments of other students by modifying her stated preference unless her assignment changes. We call the above property weak non-bossiness since we restrict the possible modification to the upper-contour-set preserving transformation.

**Definition 8** (Weak Maskin Monotonicity). *Mechanism φ satisfies weak Maskin monotonicity if for all profiles* ≻_*S*_ *and* ${≻}_{S}^{\prime }$ *such that* ${≻}_{S}^{\prime }$ *is a monotonic transformation of* ≻_*S*_ *at φ*(≻_*S*_), $\varphi_{s}\left({≻}_{S}^{\prime }\right){≻}_{s}^{\prime }\varphi_{s}\left({⪰}_{S}\right)$ *holds for each student s*.

This is a weaker version of a property called Maskin monotonicity [34], which requires that for all *s* ∈ *S*, $\varphi_{s}\left({≻}_{S}\right)=\varphi_{s}\left({≻}_{S}^{\prime }\right)$ when ${≻}_{s}^{\prime }$ is a monotonic transformation of ≻_*s*_ at *φ*_*s*_(≻_*S*_). Intuitively, Maskin monotonicity requires that the mechanism’s outcome be identical if each student *s* ranks her assigned school *φ*_*s*_(≻_*S*_) weakly higher in ${≻}_{s}^{\prime }$ compared to ≻_*s*_. We call the above property weak Maskin monotonicity since we assume $\varphi ({≻}_{S}^{\prime })$ is not necessarily identical to *φ*(≻_*S*_), although each $\varphi_{s}\left({≻}_{S}^{\prime }\right)$ must be at least as good as *φ*_*s*_(≻_*S*_), according to ${≻}_{s}^{\prime }$. A similar concept called the inconspicuous manipulation has been studied [35–37], where a student is allowed to promote exactly one school.

## 3 Family of M-convex sets

In this section, we define and illustrate the set of constraints explored throughout the paper.

### 3.1 M-convex sets

We first present the well-studied class of M-convex constraints. Assume distributional constraints are defined by a set of vectors V, i.e., $\dot{X}$ is school-feasible if $\zeta (\dot{X})\in V$. A set of distributional constraints is said to be M-convex if it can be defined as an *M-convex set*.

**Definition 9** (M-convex Set). *Let χ*_*i*_ *denote an m-element unit vector, where its i*-*th element is 1 and all other elements are 0. A set of m*-*element vectors* $V\subseteq N_{0}^{m}$ *forms an M-convex set, if for all* $\nu ,\nu^{\prime }\in V$, *for all i such that* $\nu_{i}>\nu_{i}^{\prime }$, $j\in \{k\in \left[m\right]|\nu_{k}<\nu_{k}^{\prime }\}$ *exists such that* $\nu -\chi_{i}+\chi_{j}\in V$ *and* $\nu^{\prime }+\chi_{i}-\chi_{j}\in V$ *hold*.

M-convexity is a discrete analogue of a convex set in a continuous domain. The concept of an M^♮^-convex set [38] is closely related to a convex set. An M-convex set represents a set of maximum elements within an M^♮^-convex set. Intuitively, an M-convex set does not contain any *hollow*. Definition 9 states that for any two vectors, $\nu ,\nu^{\prime }\in V$, we can find another element of V by moving one step from *ν* towards *ν*′ (i.e., $\nu -\chi_{i}+\chi_{j}\in V$), as well as by moving one step from *ν*′ toward *ν* (i.e., $\nu^{\prime }+\chi_{i}-\chi_{j}\in V$). For instance, consider a standard school choice market comprising two schools and five students, with distributional constraints forming a feasible set denoted as V={(1,4),(4,1),(2,3)}. Upon examining two feasible vectors *ν*^1^ = (1, 4) and *ν*^2^ = (4, 1), we observe that V does not satisfy the definition of M-convexity (Definition 9), since $\nu_{1}^{1}<\nu_{1}^{2}$ and $\nu_{2}^{1}>\nu_{2}^{2}$, but $\left(\nu_{1}^{2}-1,\nu_{2}^{2}+1\right)=\left(3,2\right)$ is not in V. However, by adding (3, 2) to V, we obtain a school-feasible set {(1, 4), (4, 1), (2, 3), (3, 2)} which is M-convex. The new school-feasible vector set corresponds to the distributional constraints where each school’s maximum and minimum quotas are four and one.

Kojima et al. [20] show that when the set of feasible matchings has a matroid structure [39] and the schools’ soft preferences (which aggregate the preference of each school over the students) satisfy certain easy-to-verify conditions (e.g., they can be represented as a sum of values associated with individual contracts), the Generalized Deferred Acceptance mechanism is strategyproof and fair. In our model, distributional constraints are defined on the allocation vector of $\dot{X}$, denoted as $\zeta (\dot{X})$, rather than $\dot{X}$. This distinction implies that, for each school *c*, all contracts related to it are equivalent in terms of hard distributional constraints. In contrast to Kojima et al. [20], the preference of each school over students is represented as a soft preference, which is separated from hard distributional constraints. Our model mandates that each student must be assigned to a school, and all schools are acceptable to all students, and vice versa. Consequently, the set of feasible matchings (specifically, the set of feasible contracts and their subsets) forming a matroid structure is tantamount to the set of feasible vectors V being an M-convex set.

### 3.2 Symmetric M-convex sets

The concept of symmetry is motivated by settings where schools exhibit comparable sizes, resulting in similar constraints for all schools. Thus, the set of distributional constraints is symmetric over the set of schools.

**Definition 10** (Symmetry). *Set* $V\subseteq N_{0}^{m}$ *is symmetric if for all* $\nu =(\nu_{1},\ldots ,\nu_{m})\in V$, *any permutation of* (*ν*_1_, …, *ν*_*m*_) *also belongs to* V.

We identify several distributional constraints that can be represented by a *symmetric M-convex set*. Uniform min/max quotas constraints impose minimum quotas on schools, which guarantee a minimum number of students in each school, as well as the capacity limits of schools that are generally imposed in traditional matching theory.

**Definition 11** (Uniform Min/Max Quotas Constraints). *Let p and q respectively be the minimum and maximum quotas, i.e., the number of students assigned in each school must be between p and q*. *Then the feasible set is given as* $V=\{\nu \in N_{0}^{m}|\forall i\in \left[m\right],p\leq \nu_{i}\leq q,\;\text{and}\;\sum_{i\in [m]}\nu_{i}=n\}$.

It is easy to verify that the general individual min/max quotas constraints can be represented by an M-convex set. When all the schools have identical min/max quotas, V becomes symmetric.

Introducing another class of constraints that can be represented by a symmetric M-convex set, namely, *symmetric distance constraints*, requires us to define the *most balanced vectors*.

**Definition 12** (Most Balanced Vectors). *Vector* ν∈V *is most balanced if for each i* ∈ [*m*], *ν*_*i*_ *is either* ⌊*n*/*m*⌋ *or* ⌈*n*/*m*⌉. *Let* $V^{*}$ *denote the set of all the most balanced vectors*.

**Definition 13** (Symmetric Distance Constraints). *Let* $V^{*}$ *be the set of the most balanced vectors. Given a distance threshold* d∈R, *the feasible set* V *of symmetric distance constraints is given as* $V=\{\nu \in N_{0}^{m}|\delta \left(\nu \right)\leq d,\sum_{i\in [m]}\nu_{i}=n\}$, *where δ*(*ν*) *is given as either (i) Manhattan distance* (*L*^1^ *norm*), *i.e*., ${\text{min}}_{\nu^{*}\in V^{*}}(\sum_{i\in [m]}|\nu_{i}-\nu_{i}^{*}\left|\right)$, *or (ii) Chebyshev distance* (*L*^∞^ *norm*), *i.e.*, ${\text{min}}_{\nu^{*}\in V^{*}}{\text{max}}_{i\in [m]}\left|\nu_{i}-\nu_{i}^{*}\right|$.

It is easy to verify that for a single vector *ν*, a set of vectors whose distance from *ν* is within a given threshold can be represented by an M-convex set. Since we consider distance constraints from the set of all the most balanced vectors (which is symmetric), V is also symmetric. This class of constraints is appropriate when a policymaker believes that the most balanced vectors are ideal, although she can accept any matching that is not too far from them.

### 3.3 Union of symmetric M-convex sets

In this section, we explore the class of constraints that can be represented by a *union of symmetric M-convex sets*.

#### 3.3.1 Definition and illustrative examples

First, we present a formal definition of a union of symmetric M-convex sets.

**Definition 14** (Union of Symmetric M-convex Sets). *Set* $V\subseteq N_{0}^{m}$ *is a union of symmetric M-convex sets if it is represented by* $V_{1}\cup \ldots \cup V_{\ell }$, *where for each i* ∈ [*l*], *set* $V_{i}$ *is a symmetric M-convex set*.

By its definition, the set of the unions of symmetric M-convex sets is symmetric. Thus, its intersection with the set of M-convex sets corresponds to the set of symmetric M-convex sets (Fig 1).

Several classes of constraints beyond M-convex sets can be represented by a union of symmetric M-convex sets. We first present two classes of real-life distributional constraints that can be represented by a union of symmetric M-convex sets: *ratio* and *difference* constraints. Ratio constraints specify the acceptable minimum ratio between the least and the most popular schools.

**Definition 15** (Ratio Constraints [21]). *For a given parameter α* ∈ [0, 1], V *is given as* $\left\{\nu \in N_{0}^{m}|r\left(\nu \right)\geq \alpha ,\sum_{i\in [m]}\nu_{i}=n\right\}$, *where r*(*ν*) *is given as* $\frac{{\text{min}}_{i\in [m]}\nu_{i}}{{\text{max}}_{i\in [m]}\nu_{i}}$, *i.e.*, *r*(*ν*) *is the ratio of students between the least and the most popular schools*.

Ratio constraints can be clearly decomposed into several uniform min/max quotas constraints, as Example 1 illustrates.

**Example 1**. *Assume n* = 21, *m* = 4, *and α* = 0.5. *Feasible set* V *includes* (3, 6, 6, 6), (4, 4, 5, 8), (4, 4, 6, 7), (4, 5, 5, 7), (4, 5, 6, 6), (5, 5, 5, 6), *and their permutations. It can be represented by a union of* $V_{1}$ *and* $V_{2}$, *where V*_1_ *includes* (3, 6, 6, 6), (4, 5, 6, 6), (5, 5, 5, 6), *and their permutations, and V*_2_ *includes* (4, 4, 5, 8), (4, 4, 6, 7), (4, 5, 5, 7), (4, 5, 6, 6), (5, 5, 5, 6), *and their permutations. Here V*_1_ *corresponds to the feasible set for uniform min/max quotas constraints where the minimum quota is 3 and the maximum quota is 6, and V*_2_ *orresponds to the feasible set for uniform min/max quotas constraints where the minimum quota is 4 and the maximum quota is 8*.

Difference constraints, which specify the acceptable maximum difference between the least and the most popular schools, can be interpreted as an absolute version of ratio constraints.

**Definition 16 (Difference Constraints)**. *For a given parameter* β∈R, $V=\{\nu \in N_{0}^{m}|\gamma \left(\nu \right)\leq \beta ,\sum_{i\in [m]}\nu_{i}=n\}$, *where γ*(*ν*) *is given as* max_*i*∈[*m*]_*ν*_*i*_ − min_*i*∈[*m*]_*ν*_*i*_, *i.e*., *γ*(*ν*) *is the difference between the numbers of students allocated to the most and the least popular schools*.

It is also easy to see that difference constraints can be decomposed into several uniform min/max quotas constraints, as Example 2 illustrates.

**Example 2**. *Assume n* = 21, *m* = 4, *and β* = 4. *Feasible set* V *includes* (3, 4, 7, 7), (3, 5, 6, 7), (3, 6, 6, 6), (4, 4, 5, 8), (4, 4, 6, 7), (4, 5, 5, 7), (4, 5, 6, 6), (5, 5, 5, 6), *and their permutations. It can be represented by a union of* $V_{1}$ and $V_{2}$, *where V*_1_ *includes* (3, 4, 7, 7), (3, 5, 6, 7), (3, 6, 6, 6), (4, 4, 6, 7), (4, 5, 5, 7), (4, 5, 6, 6), (5, 5, 5, 6), *and their permutations, and V*_2_ *includes* (4, 4, 5, 8), (4, 4, 6, 7), (4, 5, 5, 7), (4, 5, 6, 6), (5, 5, 5, 6), *and their permutations. Here V*_1_ *corresponds to the feasible set for uniform min/max quotas constraints where the minimum quota is 3 and the maximum quota is 7, and V*_2_ *corresponds to the feasible set for uniform min/max quotas constraints where the minimum quota is 4 and the maximum quota is 8*.

Note that even if both the ratio and difference constraints can be represented by the union of uniform min/max quotas constraints, they are not interchangeable, i.e., ratio constraints cannot be represented by difference constraints and conversely, as Proposition 1 states.

**Proposition 1**. *Ratio and difference constraints are not interchangeable*.

*Proof*. Assume *n* = 10 and *m* = 4.

First, we show that ratio constraints cannot be represented by difference constraints. Suppose *α* = 0.5. Feasible set $V_{\alpha }$ contains (2, 2, 2, 4) and all its permutations. To represent it as difference constraints, *β* must be greater than or equal to 2, but then (1, 3, 3, 3), which is not in $V_{\alpha }$, must be feasible.

Next we show that difference constraints cannot be represented by ratio constraints. Suppose *β* = 4. Feasible set $V_{\beta }$ includes (0, 3, 3, 4) and all its permutations. To represent it as ratio constraints, *α* must be 0, but then (0, 0, 0, 10), which is not in $V_{\beta }$, must be feasible.

Ratio and difference constraints constitute alternative ways to specify a well-balanced matching. Ratio constraints ensure that the ratio between the least and the most popular schools must be within interval [*α*, 1], and the difference constraints ensure that the difference between them remains within *β*. The appropriateness of each approach depends on the policymakers’ preferences and the specific applications under consideration.

We illustrate that M-convexity is not closed under the union by the following example. In particular, this example shows the union of symmetric M-convex sets is not necessarily an M-convex set.

**Example 3**. *Consider the difference constraints with n* = 10, *m* = 4, *and β* = 2. *That is, the difference in the student numbers between the most popular school and the least popular one is at most two. The school-feasible vectors can then be represented by the union of multiple symmetric M-convex sets, i.e*., $V_{1}\cup V_{2}$, *where* $V_{1}$ *consists of* (1, 3, 3, 3), (2, 2, 3, 3) *and all their permutations, and* $V_{2}$ *consists of* (2, 2, 2, 4), (2, 2, 3, 3), *and all their permutations. We consider the following two vectors*: $\nu =\left(1,3,3,3\right)\in V_{1}$ *and* $\nu^{\prime }=\left(2,2,2,4\right)\in V_{2}$. $\nu_{2}>\nu_{2}^{\prime }$ *holds. For both j* = 1 *and j* = 4 *where* $\nu_{j}<\nu_{j}^{\prime }$ *holds, the condition of M-convexity is not satisfied by this set of feasible vectors. For j* = 1, *ν*′ + *χ*_2_ − *χ*_1_ = (1, 3, 2, 4) *is not school-feasible, and for j* = 4, *ν* − *χ*_2_ + *χ*_4_ = (1, 2, 3, 4) *is not school-feasible, since the required difference excesses β*.

Next we present another type of union of symmetric M-convex constraints obtained by combining several symmetric M-convex constraints. If a policymaker believes that several symmetric M-convex constraints are equally appropriate, she can define the union of these vectors as school-feasible. This situation assumes that all constraints are equally justifiable and that satisfying one is necessary for feasibility. For illustration, by combining uniform min/max and distance constraints, we obtain the following class of constraints, which we call *flexible uniform min/max quotas constraints*.

**Definition 17** (Flexible Uniform Min/Max Quotas Constraints). *Let p and q respectively be the minimum and maximum quotas. Let* d∈R *be the distance threshold, and let* $V^{*}$ *be the set of the most balanced vectors. Then* $V=\left\{\nu \in N_{0}^{m}|\forall i\in \left[m\right],p\leq \nu_{i}\leq q\;\text{and}\;\sum_{i\in [m]}\nu_{i}=n\right\}⋃\left\{\nu \in N_{0}^{m}|\delta \left(\nu \right)\leq d\;\text{and}\;\sum_{i\in [m]}\nu_{i}=n\right\}$, *where δ*(*ν*) *is given as either (i) Manhattan distance* (*L*^1^ *norm*), ${\text{min}}_{\nu^{*}\in V^{*}}(\sum_{i\in [m]}|\nu_{i}-\nu_{i}^{*}\left|\right)$, *or (ii) Chebyshev distance* (*L*^∞^ *norm*), ${\text{min}}_{\nu^{*}\in V^{*}}{\text{max}}_{i\in [m]}\left|\nu_{i}-\nu_{i}^{*}\right|$.

Notice that, as illustrated in Fig 1, components of the unions of symmetric M-convex sets, i.e., the class of symmetric M-convex sets, is a subclass of M-convex sets, while the two classes, the union of symmetric M-convex sets and M-convex set, are not included by each other. We use the following example to show the intuition.

**Example 4** (Example 1 continued). *We show by reusing Example 1, in which the ratio-constraint feasible set* V *is the union of symmetric M-convex sets* $V_{1}$ *and* $V_{2}$. *Taking* $V_{1}$ *(uniform min/max quotas of 3/6) as an instance, we can easily extend it by re-specifying the min/max quotas of c*_1_ *(the first element of feasible vectors) as 3/7 instead of 3/6. Then we obtain an asymmetric M-convex set. Moreover, notice that* V, *a union of two symmetric M-convex sets, is not M-convex. Observe that ν* = (3, 6, 6, 6) *and ν*′ = (4, 4, 5, 8) *are in* V, *and* $\nu_{4}^{\prime }>\nu_{4}$ *and* $\nu_{2}^{\prime }<\nu_{2}$. *However, by subtracting 1 from ν*_2_ *and adding 1 to ν*_4_, *we obtain* (3, 5, 6, 7), *which is not in* V.

Note that Definition 14 includes the case that V is represented by just one symmetric M-convex set. Thus, all results in this paper also hold for a symmetric M-convex set.

#### 3. 3.2 Impossibility results

Under distributional constraints, fairness and nonwastefulness are generally incompatible, especially under the union of symmetric M-convex sets. Yahiro et al. [21] show that these two notions are incompatible under ratio constraints, which is a special case of the union of symmetric M-convex constraints. We strengthen this result by showing that fairness and nonwastefulness are incompatible under difference constraints.

**Proposition 2**. *Under difference constraints, fairness and nonwastefulness are incompatible*.

*Proof*. Consider the following example (Example 5), to which the example used in Yahiro et al. [21] is trivially extended in the context of difference constraints.

**Example 5** (Adapted from [21]). *Consider S* = {*s*_1_, *s*_2_, *s*_3_, *s*_4_} and *C* = {*c*_1_, *c*_2_, *c*_3_}, *β* = 1. *The following are the preferences of the students and the schools*:

$$\begin{array}{c} \begin{array}{c} s_{1},s_{2}:c_{2}≻c_{3}≻c_{1}\;\\ s_{3},s_{4}:c_{3}≻c_{2}≻c_{1}\; \end{array}\begin{array}{c} c_{1}:s_{1}≻s_{3}≻s_{2}≻s_{4}\\ c_{2}:s_{2}≻s_{3}≻s_{1}≻s_{4}\\ c_{3}:s_{4}≻s_{1}≻s_{3}≻s_{2}. \end{array} \end{array}$$

Since *β* = 1, the difference in the number of students between the most and the least popular schools should be smaller or equal to 1. Hence, the matchings that satisfy these constraints are such that one school is assigned two students and each of the other two schools is assigned one student. If a feasible matching is fair, it must contain (*s*_2_, *c*_2_) and (*s*_4_, *c*_3_); otherwise, either *s*_2_ or *s*_4_ has justified envy. Here, although *c*_1_ is the least preferred school for each student, at least one student must be assigned to it. Assigning both *s*_1_ and *s*_3_ to *c*_1_ is wasteful. Assume we assign *s*_1_ to *c*_1_. If we assign *s*_3_ to *c*_2_, *s*_3_ claims an empty seat in *c*_3_. If we assign *s*_3_ to *c*_3_, *s*_1_ has justified envy toward *s*_3_. Thus assume we assign *s*_3_ to *c*_1_. If we assign *s*_1_ to *c*_3_, *s*_1_ claims an empty seat in *c*_2_. If we assign *s*_1_ to *c*_2_, *s*_3_ has justified envy toward *s*_1_.

Example 5 also shows that fairness and nonwastefulness are incompatible under a symmetric M-convex constraints since it includes only (1, 1, 2) and its permutations, which can be represented by a single symmetric M-convex set. Since fairness and nonwastefulness are incompatible under the union of symmetric M-convex constraints, we focus on *finding a fair outcome while reducing as much wastefulness as possible*. Observe also that nonwastefulness is weaker than Pareto optimality (recall Definition 6), and therefore, fairness is also incompatible with Pareto optimality under the union of symmetric M-convex constraints.

As described earlier, according to Kojima et al. [20], if the set of feasible vectors forms an M-convex set, then a generalized mechanism is strategyproof and fair. However, we cannot apply it to a union of symmetric M-convex sets since it is not an M-convex set as illustrated in Example 3.

Beyond M-convex, identifying constraints, for which we can develop a strategyproof and fair mechanism, is a challenging question. In this paper, we identify a general class of constraints beyond M-convex sets, namely, the union of symmetric M-convex sets, for which a strategyproof and fair mechanism can be developed.

### 3.4 Theoretical properties

Now we present two properties that are satisfied by a union of symmetric M-convex constraints. They are crucial for analyzing our mechanism.

**Lemma 1**. *If* $\nu =(\nu_{1},\ldots ,\nu_{i},\ldots ,\nu_{j},\ldots ,\nu_{m})\in V$ *where* V *is a union of symmetric M-convex sets and ν*_*i*_ > *ν*_*j*_ *holds, then vector ν*′ = (*ν*_1_, …, *ν*_*i*_ − 1, …, *ν*_*j*_ + 1, …, *ν*_*m*_) *and all of its permutations also belong to* V.

*Proof*. From symmetry, vector *ν*′′, obtained by exchanging the *i*-th and *j*-th elements of *ν*, is also feasible. Then by the definition of an M-convex set, since $\nu_{i}>\nu_{i}^{\prime \prime }$, $\nu_{j}<\nu_{j}^{\prime \prime }$, and $\nu_{k}=\nu_{k}^{\prime \prime }$ for all *k* ≠ *i*, *j*, vector *ν* − *χ*_*i*_ + *χ*_*j*_ = *ν*′ must be in V.

Intuitively, Lemma 1 states that when transforming a feasible vector *ν* into a more balanced vector *ν*′, feasibility is preserved. This idea leads to the following lemma about most balanced vectors.

**Lemma 2**. *If* V, where V≠∅, *is a union of symmetric M-convex set, it contains all of the most balanced vectors*.

*Proof*. Assume first that V is a symmetric M-convex set, and let *ν* be a vector in V. If *ν* is most balanced, then from symmetry, all of its permutations must belong to V. Thus, V contains all of the most balanced vectors. If *ν* is not most balanced, from Lemma 1, we can move one student from a popular school to a less popular school in *ν* and obtain another vector included in V. By repeatedly applying such modification, we eventually obtain a most balanced vector that belongs to V.

Therefore, if V is a union of symmetric M-convex sets, it includes all the most balanced vectors.

## 4 Mechanism: Quota reduction deferred acceptance

In this section, we introduce the Quota Reduction Deferred Acceptance mechanism (QRDA) and present its axiomatic properties. We theoretically compare it with a baseline mechanism.

### 4.1 Mechanism description

We first introduce the standard Deferred Acceptance mechanism (DA), which is a component of our mechanism. Standard DA is defined with a standard market. A *standard market* is a tuple (*S*, *C*, ≻_*S*_, ≻_*C*_, *q*_*C*_), whose definition resembles a market with distributional constraints. The only difference is that its constraints are given as a profile of individual maximum quotas: *q*_*C*_ = (*q*_*c*_)_*c* ∈ *C*_. Matching $\dot{X}$ is school-feasible if for all *c* ∈ *C*, $|{\dot{X}}_{c}|\leq q_{c}$ holds. Standard DA is defined as follows.

**Mechanism 1 (Standard DA)**

**Step 1** *Each student s applies to her favorite school, according to* ≻_*s*_, *among the schools that have not rejected her so far*.
**Step 2** *Each school c provisionally accepts the top q*_*c*_ *students from the applying students based on* ≻_*c*_ *and rejects the rest of them without any distinctions between newly applying and already provisionally accepted students*.
**Step 3** *Barring any rejected students, return the current matching. Otherwise, go to **Step 1***.

To develop a DA-based mechanism on distributional constraints of union of symmetric M-convex sets, the crux is to specify the appropriate maximum quotas, by using the standard DA which can always be guaranteed to output feasible matchings. We explore this idea by utilizing flexible maximum quotas, such that we can enhance efficiency while guaranteeing feasibility and other desired properties. We also compare our proposed mechanism with another, which uses a fixed way to determine the maximum quotas, later in Section 4.3.

Informally, QRDA produces an initial standard market from a market with distributional constraints and then, at each stage, it iteratively (i) restricts the constraints on this market (i.e., reduces the maximum quotas) and (ii) applies DA to it until the matching returned by DA is also feasible with respect to the distributional constraints.

Which school’s maximum quota is reduced at the beginning of each stage is defined by *σ*, a sequence of schools based on the round-robin order *c*_1_, *c*_2_, …, *c*_*m*_. Let *σ*(*k*) denote the *k*-th school in *σ*, i.e., *σ*(*k*) = *c*_*j*_, where j=1+(k-1modm). For simplicity, although we assume *σ* is based on a fixed round-robin order, all our results hold for any *balanced* sequence *σ*. A sequence *σ* is said to be *balanced* if for each $\ell \in N_{0}$, *σ*(*m*ℓ + 1), *σ*(*m*ℓ + 2), …, *σ*(*m*ℓ + *m*) is a permutation of *c*_1_, *c*_2_, …, *c*_*m*_. The balanced sequence requirement is crucial to guarantee the strategyproofness of QRDA, as Example 7 later demonstrates.

QRDA is defined with respect to a specific quota reduction sequence *σ*. However, in the following, for simplicity, we assume that *σ* is based on the round-robin order *c*_1_, *c*_2_, …, *c*_*m*_. When necessary, we specify *σ* and denote QRDA based on *σ* by QRDA^*σ*^.

Let *ν*^*max*^ be a value that satisfies $\forall \nu \in V,\forall i\in \left[m\right],\nu^{max}\geq \nu_{i}$. Also, let $q_{c}^{k}$ denote the quota of school *c* at stage *k* of QRDA. Given *σ* and *ν*^*max*^, QRDA is defined.

**Mechanism 2 (QRDA)**

**Initialization:**
  *For all c* ∈ *C*, $q_{c}^{1}\leftarrow \nu^{max}$.
**Initial Stage:**
  **Step 1** *Run standard DA in market* $\left(S,C,{≻}_{S},{≻}_{C},q_{C}^{1}\right)$ and obtain matching ${\dot{X}}^{1}$.
  **Step 2** *If* ${\dot{X}}^{1}$ *is school-feasible, then return* ${\dot{X}}^{1}$ . *Otherwise, go to **Stage** 2*.
**Stage *k* (≥2):**
  **Step 1** *For school c*′ = *σ*(*k* − 1), $q_{c^{\prime }}^{k}\leftarrow q_{c^{\prime }}^{k-1}-1$, *and for c* ≠ *c*′, $q_{c}^{k}\leftarrow q_{c}^{k-1}$.
  **Step 2** *Run standard DA in market* $\left(S,C,{≻}_{S},{≻}_{C},q_{C}^{k}\right)$ *and obtain matching* ${\dot{X}}^{k}$ .
  **Step 3** *If* ${\dot{X}}^{k}$ *is school-feasible, then return* ${\dot{X}}^{k}$ . *Otherwise, go to **Stage**k* + 1.

Note that in the following arguments, especially in Theorem 7, we assume that it only costs constant time to verify whether a matching is feasible or not. Also, note that the complexity/cost for finding ${\text{max}}_{\nu \in V,i\in [m]}\nu_{i}$ depends on the representation of feasible vectors V, which can be high. However, we only require that *ν*^*max*^ is not smaller than ${\text{max}}_{\nu \in V,i\in [m]}\nu_{i}$. Thus, we can always choose *ν*^*max*^ = *n*. The choice of *ν*^*max*^ does not influence the complexity result in Theorem 7.

### 4.2 Mechanism properties

First we show that QRDA always returns a feasible and fair matching by Theorem 1.

**Theorem 1**. *QRDA returns a feasible and fair matching when* V *is a union of symmetric M-convex sets*.

*Proof*. If QRDA returns a matching, it is clearly feasible. According to Lemma 2, all the most balanced vectors must be included in V. Eventually, at some stage *k*′, the quota of each school equals either ⌊*n*/*m*⌋ or ⌈*n*/*m*⌉, and $\sum_{i\in [m]}q_{c_{i}}^{k^{\prime }}=n$. At this stage, standard DA returns a feasible matching. Thus, QRDA must terminate at an earlier stage *k* (≤ *k*′) by returning a feasible matching. The matching returned at stage *k* is identical to the one returned by standard DA for the market $\left(S,C,{≻}_{S},{≻}_{C},q_{C}^{k}\right)$. Therefore, since standard DA is fair [18], QRDA is also fair.

Actually, this theorem holds under a much weaker condition; the fact that most balanced vectors are included in V is sufficient.

Now we show that QRDA is strategyproof. Although using DA iteratively, QRDA does not trivially inherit strategyproofness from DA. Since the quotas of schools are decreasing, a student might have an incentive to terminate the mechanism early to secure the school seat, which might not be available at later stages. Therefore, the quotas have to be reduced carefully to preserve strategyproofness, i.e., the quota reduction sequence has to be balanced.

Moreover, under general non-M-convex constraints, a balanced quota reduction sequence may not be sufficient to preserve the strategyproofness of iterative DA mechanisms. Yahiro et al. [21] provided an example illustrating this fact, which we reproduce with Example 6 for the sake of completeness. In this example, initial quotas are equal to the largest number of students in a school in any feasible matching and the quota reduction sequence is balanced.

**Example 6** ([21]). *Consider S* = {*s*_1_, *s*_2_, *s*_3_, *s*_4_, *s*_5_, *s*_6_}, *C* = {*c*_1_, *c*_2_, *c*_3_}, *σ*: *c*_1_, *c*_2_, *c*_3_, and V={(3,1,2),(2,2,2)}. *The following are the preferences of the students and the schools*:

$$\begin{array}{c} \begin{array}{c} s_{1},s_{2}:c_{1}≻c_{2}≻c_{3}\;\\ s_{3}:c_{2}≻c_{3}≻c_{1}\;\\ s_{4},s_{5},s_{6}:c_{3}≻c_{1}≻c_{2}\; \end{array}\begin{array}{c} c_{1},c_{2},c_{3}:s_{1}≻s_{2}≻s_{3}≻s_{4}≻s_{5}≻s_{6}. \end{array} \end{array}$$

The initial maximum quotas are $q_{C}^{1}=\left(3,3,3\right)$. At stage 1, all the students are assigned to their favorite school, and the matching is not feasible. The mechanism proceeds by reducing the quota of schools *c*_1_ and *c*_2_ by one at stages 2 and 3, and the matching remains the same. At stage 4, the quota of school *c*_3_ is decreased by one. Student *s*_6_ is rejected and then applies to *c*_1_, which also rejects her. Hence, *s*_6_ is assigned to *c*_2_, and the matching becomes feasible. However, if *s*_6_ misreports her preference with ${≻}_{s_{6}}^{\prime }$ such that *c*_1_ is her favorite school, she is assigned to *c*_1_ at stage 1 and the matching is feasible. Thus, *s*_6_ can successfully manipulate the mechanism.

Hence, QRDA’s strategyproofness is not trivial, and to prove it, we develop novel proof techniques, which require several lemmas.

The first is called the *Scenario lemma* and it requires two definitions. A scenario is a sequence of schools to which a student plans to apply. For example, suppose that student *s* has scenario $C_{s}$, defined as *c*_1_, *c*_2_, *c*_3_. This scenario $C_{s}$ means that student *s* first applies to school *c*_1_, and if she is rejected, then she applies to school *c*_2_, and then to *c*_3_ if she is rejected again. A scenario is not necessarily exhaustive. A *rejection chain* $R(C_{s})$, based on scenario $C_{s}$, is the sequence of all the students and the schools’ actions (applications and rejections) that follow when student *s* enters the market with scenario $C_{s}$, starting when she applies to the first school of this scenario. A rejection chain ends when (i) student *s* is rejected by the last school in $C_{s}$ or (ii) the mechanism terminates. A simple example of a rejection chain is presented in Table 2.

**Table 2:** Example of a rejection chain.

Stage	#	Action
*k*	1	Student *s* applies to school *c*_1_.
	2	School *c*_1_ rejects student *s*_1_.
	3	Student *s*_1_ applies to school *c*_2_ (and is accepted).
*k* + 1	1	School *c*_3_ rejects student *s*_2_ (due to its quota reduction).
	2	Student *s*_2_ applies to school *c*_4_.
		…

Given these definitions, we can define the Scenario lemma, which is inspired by the original Scenario lemma [40]. It was proven in the context of ratio constraints [21], and the proof is trivially extended to constraints represented by a union of symmetric M-convex sets.

**Lemma 3** (Scenario Lemma). *Consider two scenarios*, $C_{s}$ *and* $C_{s}^{\prime }$, *of student s starting from the same stage of QRDA. If (1) each school that appears in* $C_{s}^{\prime }$ *also appears in* $C_{s}$ *(the order is irrelevant), (2) student s applies to all the schools in* $C_{s}$, *and (3) all the actions of* $R(C_{s}^{\prime })$ *happen at the same stage, then all the actions in* $R(C_{s}^{\prime })$ *also happen in* $R(C_{s})$.

*Proof*. The first action in $R(C_{s}^{\prime })$ is “student *s* applies to school *c*,” where *c* is the first school that appears in $C_{s}^{\prime }$. Since *c* also appears in $C_{s}$, and *s* applies to all the schools in $C_{s}$, $R(C_{s})$ also includes this action. For an inductive step, assume the first *i* − 1 actions in $R(C_{s}^{\prime })$ also happen in $R(C_{s})$, and consider the *i*-th action of $R(C_{s}^{\prime })$. The *i*-th action in $R(C_{s}^{\prime })$ must be either (i) “student *s*′ applies to school *c*′” or (ii) “school *c*′ rejects student *s*′.”

In case (i) with *s*′ = *s*, since school *c*′ must appear in $C_{s}$ and *s* applies to all the schools in $C_{s}$, $R(C_{s})$ also includes this action. In case (i) with *s*′ ≠ *s*, there must be a previous action, “school *c*′′ rejects student *s*′,” in $R(C_{s}^{\prime })$. From the inductive assumption, this action also happens in $R(C_{s})$. Thus, the action “student *s*′ applies to school *c*′” also happens in $R(C_{s})$.

In case (ii), let $S_{c^{\prime }}^{\prime }$ be the set of students who applied to *c*′ before the *i*-th action in $R(C_{s}^{\prime })$, and let *S*_*c*′_ be the set of all the students applying to *c*′ until all actions in $R(C_{s})$ are executed. Here, $S_{c^{\prime }}^{\prime }\subseteq S_{c^{\prime }}$ holds since every application before the *i*-th action in $R(C_{s}^{\prime })$ also appears in $R(C_{s})$. Since in the *i*-th action of $R(C_{s}^{\prime })$, *s*′ is rejected by school *c*′, she is not among *c*′’s favorite $q_{c^{\prime }}^{k}$ students in set $S_{c^{\prime }}^{\prime }$. Since the quotas of schools are non-increasing as QRDA continues, in some stage *k*′ (≥*k*), student *s*′ must not be among the favorite $q_{c^{\prime }}^{k^{\prime }}$ students in *S*_*c*′_. Thus, the action “school *c*′ rejects student *s*′” eventually occurs in $R(C_{s})$.

Another essential lemma for QRDA’s strategyproofness in our setting is Lemma 4. Before introducing it, we introduce the following notation and concepts. Given a matching $\dot{X}$, consider that some school *c*_*i*_’s student number is decreased by one and another school *c*_*j*_’s student number is increased by one. We denote by *η* the corresponding *operation*, i.e., the operation that transforms vector $\left(|{\dot{X}}_{c_{1}}|,\ldots ,|{\dot{X}}_{c_{i}}|,|{\dot{X}}_{c_{j}}|,\ldots ,|{\dot{X}}_{c_{m}}|\right)$ into vector $\left(|{\dot{X}}_{c_{1}}|,\ldots ,|{\dot{X}}_{c_{i}}|-1,|{\dot{X}}_{c_{j}}|+1,\ldots ,|{\dot{X}}_{c_{m}}|\right)$. Moreover, at stage *k* of QRDA, we denote by *η*_*k*_ the operation that transforms vector $\zeta ({\dot{X}}^{k-1})$ into vector $\zeta ({\dot{X}}^{k})$ since at most one student number changes in a QRDA’s stage. Finally, at stage *k* of QRDA, a school *c* is said to be *full* if $|{\dot{X}}_{c}^{k}|=q_{c}^{k}$, and *maximum* if $|{\dot{X}}_{c}^{k}|={\text{max}}_{c^{\prime }\in C}\left|{\dot{X}}_{c^{\prime }}^{k}\right|$. Note that *c*_*i*_ is always full and maximum, and *c*_*j*_ is neither full nor maximum in QRDA since *σ* is a balanced sequence.

Intuitively, Lemma 4 means that, at some stage of QRDA, if operation *η* exists that makes the current QRDA’s matching feasible, at any subsequent stage of QRDA, this operation (more specifically, any operation that resembles *η*, as defined in Lemma 4) also makes the current QRDA’s matching feasible.

**Lemma 4**. *Consider an infeasible matching* ${\dot{X}}^{k}$ *obtained at stage k of QRDA and an operation η that leads to a feasible matching when applied to* ${\dot{X}}^{k-1}$. *Assume that operation η increases school c*_*j*_’*s student number, and let k*′ *denote the first stage of QRDA after k* (<*k*′) *where c*_*j*_’*s student number increases. Then at any stage* $\hat{k}$ *such that* $k-1\leq \hat{k}<k^{\prime }$ *and for any maximum school c*_*max*_ *at stage* $\hat{k}$, *applying an operation that reduces c*_*max*_’*s student number and increases c*_*j*_’*s student number to matching* ${\dot{X}}^{\hat{k}}$ *leads to a feasible matching*.

*Proof*. Consider an infeasible matching ${\dot{X}}^{k}$ obtained at stage *k* of QRDA and an operation *η* such that imposing *η* on $\zeta ({\dot{X}}^{k-1})$ leads to a feasible vector. Assume that operation *η* reduces school *c*_*i*_’s student number by one and increases school *c*_*j*_’s student number by one. The proof is done by induction.

For $\hat{k}=k-1$, vector $\left(\ldots ,|{\dot{X}}_{c_{i}}^{k-1}|-1,|{\dot{X}}_{c_{j}}^{k-1}|+1,\ldots \right)$ is feasible by assumption. If school *c*_*i*_ is the unique maximum school at stage *k* − 1, then the claim is satisfied for $\hat{k}=k-1$. Hence, consider a maximum school *c*_*max*_ ≠ *c*_*i*_ at stage *k* − 1. By definition, $|{\dot{X}}_{c_{max}}^{k-1}\left|\geq \right|{\dot{X}}_{c_{i}}^{k-1}|$, which implies $|{\dot{X}}_{c_{max}}^{k-1}\left|>\right|{\dot{X}}_{c_{i}}^{k-1}|-1$, and thus with Lemma 1 on $\left(\ldots ,|{\dot{X}}_{c_{i}}^{k-1}|-1,|{\dot{X}}_{c_{j}}^{k-1}|+1,\ldots \right)$, vector $\left(\ldots ,|{\dot{X}}_{c_{max}}^{k-1}|-1,|{\dot{X}}_{c_{j}}^{k-1}|+1,\ldots \right)$ is feasible.

Consider some stage $\hat{k}$ such that $k-1<\hat{k}<k^{\prime }$ and assume that the claim is true at stage $\hat{k}-1$. We show that the claim is also true for $\hat{k}$. Assume that operation $\eta_{\hat{k}}$ reduces school *c*_*a*_’s student number and increases school *c*_*b*_’s student number. Since school *c*_*a*_ is maximum at stage $\hat{k}-1$ and the claim is true at stage $\hat{k}-1$, vector $\left(\ldots ,|{\dot{X}}_{c_{a}}^{\hat{k}-1}|-1,|{\dot{X}}_{c_{j}}^{\hat{k}-1}|+1,\ldots \right)$ is feasible. Note that if *c*_*b*_ = *c*_*j*_ then $\zeta ({\dot{X}}^{\hat{k}})$ is feasible and QRDA terminates strictly before *k*′, which is a contradiction. Hence, in the following, we assume *c*_*b*_ ≠ *c*_*j*_. Two cases are possible.

If $|{\dot{X}}_{c_{a}}^{\hat{k}-1}\left|-1=\right|{\dot{X}}_{c_{b}}^{\hat{k}-1}|$, then vector $\zeta ({\dot{X}}^{\hat{k}})$ is a permutation of vector $\zeta ({\dot{X}}^{\hat{k}-1})$. Hence, since *c*_*b*_ ≠ *c*_*j*_, for any maximum school *c*_*max*_ at $\hat{k}$, vector $\left(\ldots ,|{\dot{X}}_{c_{max}}^{\hat{k}}|-1,|{\dot{X}}_{c_{j}}^{\hat{k}}|+1,\ldots \right)$ is a permutation of vector $\left(\ldots ,|{\dot{X}}_{c_{a}}^{\hat{k}-1}|-1,|{\dot{X}}_{c_{j}}^{\hat{k}-1}|+1,\ldots \right)$. Hence, by symmetry, vector $\left(\ldots ,|{\dot{X}}_{c_{max}}^{\hat{k}}|-1,|{\dot{X}}_{c_{j}}^{\hat{k}}|+1,\ldots \right)$ is feasible.

Otherwise, $|{\dot{X}}_{c_{a}}^{\hat{k}-1}\left|-1>\right|{\dot{X}}_{c_{b}}^{\hat{k}-1}|$. By Lemma 1 on vector $\left(\ldots ,|{\dot{X}}_{c_{a}}^{\hat{k}-1}|-1,|{\dot{X}}_{c_{j}}^{\hat{k}-1}|+1,\ldots \right)$, we obtain that vector $\left(\ldots ,|{\dot{X}}_{c_{a}}^{\hat{k}-1}|-2,|{\dot{X}}_{c_{j}}^{\hat{k}-1}|+1,|{\dot{X}}_{c_{b}}^{\hat{k}-1}|+1\ldots \right)$ is feasible, i.e., vector $\left(\ldots ,|{\dot{X}}_{c_{a}}^{\hat{k}}|-1,|{\dot{X}}_{c_{j}}^{\hat{k}}|+1,|{\dot{X}}_{c_{b}}^{\hat{k}}|\ldots \right)$ is feasible. If school *c*_*a*_ is the unique maximum school at stage $\hat{k}$, then the claim is satisfied at $\hat{k}$. Hence, consider a maximum school *c*_*max*_ ≠ *c*_*a*_ at $\hat{k}$. By definition, $|{\dot{X}}_{c_{max}}^{\hat{k}}\left|\geq \right|{\dot{X}}_{c_{a}}^{\hat{k}}|$, which implies $|{\dot{X}}_{c_{max}}^{\hat{k}}\left|>\right|{\dot{X}}_{c_{a}}^{\hat{k}}|-1$, and thus with Lemma 1 on $\left(\ldots ,|{\dot{X}}_{c_{a}}^{\hat{k}}|-1,|{\dot{X}}_{c_{j}}^{\hat{k}}|+1,\ldots \right)$, vector $\left(\ldots ,|{\dot{X}}_{c_{max}}^{\hat{k}}|-1,|{\dot{X}}_{c_{j}}^{\hat{k}}|+1,\ldots \right)$ is feasible, which concludes the proof.

Now we are ready to show that QRDA is strategyproof under constraints represented by a union of symmetric M-convex sets.

**Theorem 2**. *QRDA is strategyproof when* V *is a union of symmetric M-convex sets*.

*Proof*. By contradiction, assume student *s* is assigned to a better school when she misreports. Without loss of generality, we assume that her true preference is *c*_1_ ≻_*s*_ *c*_2_ ≻_*s*_ …≻_*s*_*c*_*m*_, and that *s* is assigned to school *c*_*j*_ in stage *k* when misreporting, but *s* is assigned to *c*_*i*_ at stage *k*′ under *s*’s true preference, where *j* < *i*.

First, we show that if *k*′ ≤ *k*, student *s* cannot benefit from misreporting. The standard DA satisfies a property called resource monotonicity, i.e., DA’s outcome is weakly less preferred by each student if the quotas decrease [41]. This property implies that when student *s* truthfully reports her preference at both stages *k* and *k*′, her assignment is (weakly) worse in *k* than in *k*′. Furthermore, DA is strategyproof [40, 42]. Hence, at stage *k*, student *s*’s assignment is worse when she misreports than when she truthfully reports. Therefore, *s*’s assignment is (weakly) worse when she misreports in *k* than when she truthfully reports in *k*′, and thus, *s* cannot benefit from misreporting if *k*′ ≤ *k*. Hence, in the following, *k* < *k*′ holds.

At stage *k*, consider an alternative (and equivalent) DA mechanism where we run a standard DA for *S* \ {*s*} under the quotas of stage *k* and add *s* to the market. The matching obtained in this way is identical to the matching obtained by applying DA when all the students enter the market simultaneously [40]. When *s* is added to the market with a misreported preference, *s* is assigned to school *c*_*j*_ and a feasible matching ${\ddot{X}}^{k}$ is obtained; when *s* is added with a true preference, *s* is assigned to school *c*_*o*_, and an infeasible matching ${\dot{X}}^{k}$ is obtained.

Now we define two scenarios: (a) $C_{s}=\left(c_{1},c_{2},\ldots ,c_{i-1}\right)$, based on the true preference of *s*, and so the last action in $R(C_{s})$ must be “school *c*_*i*−1_ rejects student *s*,” and (b) $C_{s}^{\prime }$, based on the misreported preferences in which the last school is *c*_*j*_. For $C_{s}^{\prime }$, the following two cases are possible: (i) *c*_*j*_ is truly the least preferred school for *s* within $C_{s}^{\prime }$ or (ii) $C_{s}^{\prime }$ contains at least one school that is less preferred by *s* than *c*_*j*_. We start by proving case (i); case (ii) is proven in the last paragraph of the proof. A key point in case (i) is that each school *c* that appears in $C_{s}^{\prime }$ also appears in $C_{s}$. Thus by Lemma 3, it implies that action “student *s* applies to school *c*_*j*_” appears in rejection chain $R(C_{s})$.

At stage *k*, when *s* enters the market (after all the other students) with true preferences, assume that school *c*_*e*_ gains one student, and when *s* misreports, another school *c*_*d*_ gains one student. We show the proof for a particular case when *c*_*e*_ = *c*_*o*_ and *c*_*d*_ = *c*_*j*_; a similar argument can be developed when *c*_*e*_ ≠ *c*_*o*_ or *c*_*d*_ ≠ *c*_*j*_ by replacing *c*_*o*_ to *c*_*e*_, and *c*_*j*_ to *c*_*d*_, respectively, in the following argument.

Note that $|{\ddot{X}}_{c_{o}}^{k}\left|=\right|{\dot{X}}_{c_{o}}^{k}|-1$, $|{\ddot{X}}_{c_{j}}^{k}\left|=\right|{\dot{X}}_{c_{j}}^{k}|+1$, and for all other schools *c* (≠ *c*_*o*_, *c*_*j*_), $|{\ddot{X}}_{c}^{k}\left|=\right|{\dot{X}}_{c}^{k}|$. Therefore, the vector of feasible matching ${\ddot{X}}^{k}$ verifies $\zeta \left({\ddot{X}}^{k}\right)=\left(\right|{\dot{X}}_{c_{1}}^{k}\left|,\ldots ,\right|{\dot{X}}_{c_{o}}^{k}\left|-1,\ldots ,\right|{\dot{X}}_{c_{j}}^{k}\left|+1,\ldots ,\right|{\dot{X}}_{c_{m}}^{k}\left|\right)$.

It follows that if $|{\dot{X}}_{c_{o}}^{k}\left|-1<\right|{\dot{X}}_{c_{j}}^{k}|+1$ holds, then by Lemma 1 on $\zeta ({\ddot{X}}^{k})$, ${\dot{X}}^{k}$ is also feasible, which is a contradiction. Thus we obtain the following equation:

$$\begin{array}{c} |{\dot{X}}_{c_{o}}^{k}\left|\geq \right|{\dot{X}}_{c_{j}}^{k}|+2. \end{array}$$ (1)

The rest of the proof for case (i) is composed of three arguments: (A), (B), and (C).

(A) First, we show that under scenario $C_{s}$, school *c*_*j*_’s student number does not increase strictly between *k* and *k*′. By contradiction, assume that *c*_*j*_’s student number increases at some stage $\tilde{k}$ ($k<\tilde{k}<k^{\prime }$). Let *c*_⋆_ denote a school whose student number is decreased by operation $\eta_{\tilde{k}}$. Since school *c*_⋆_ is maximum at stage $\tilde{k}-1$, by Lemma 4 with $\hat{k}\leftarrow \tilde{k}-1$, vector $\left(\ldots ,|{\dot{X}}_{c_{⋆}}^{\tilde{k}-1}|-1,|{\dot{X}}_{c_{j}}^{\tilde{k}-1}|+1,\ldots \right)$ is feasible, i.e., vector $\zeta ({\dot{X}}^{\tilde{k}})$ is feasible. Hence, QRDA terminates strictly before *k*′, which is a contradiction.

(B) Recall that under scenario $C_{s}$, action “student *s* applies to school *c*_*j*_” must occur; however, in this scenario student *s*’s final assignment is *c*_*i*_ (≠ *c*_*j*_). Thus, school *c*_*j*_ rejects student *s* at some stage of QRDA. Notice that if school *c*_*j*_ is not full at stage *k*′ − 1, then *c*_*j*_ cannot reject any students at stage *k*′ or at any previous stage. Hence, school *c*_*j*_ is full at stage *k*′ − 1, i.e., $q_{c_{j}}^{k^{\prime }-1}=\left|{\dot{X}}_{c_{j}}^{k^{\prime }-1}\right|$, which implies that for any school *c*, $|{\dot{X}}_{c_{j}}^{k^{\prime }-1}\left|+1\geq \right|{\dot{X}}_{c}^{k^{\prime }-1}|$. Since school *c*_*j*_’s student number does not increase strictly between *k* and *k*′, the following equation holds for any school *c*:

$$\begin{array}{c} |{\dot{X}}_{c_{j}}^{k}\left|+1\geq \right|{\dot{X}}_{c}^{k^{\prime }-1}|. \end{array}$$ (2)

Let $\hat{k}$ denote the first stage after *k* such that $|{\dot{X}}_{c_{j}}^{k}\left|+1\geq \right|{\dot{X}}_{c}^{\hat{k}}|$ for any school *c*. With Eqs 1 and 2, we know that $k<\hat{k}\leq k^{\prime }-1$. Assume that operation $\eta_{\hat{k}}$ decreases *c*_*a*_’s student number and increases *c*_*b*_. By the definition of stage $\hat{k}$, $|{\dot{X}}_{c_{a}}^{\hat{k}-1}\left|=\right|{\dot{X}}_{c_{j}}^{k}|+2$ and $|{\dot{X}}_{c_{b}}^{\hat{k}-1}\left|<\right|{\dot{X}}_{c_{j}}^{k}|+1$ hold.

(C) Finally, we show that vector $\zeta ({\dot{X}}^{\hat{k}})$ is feasible. Since school *c*_*a*_ is maximum at stage $\hat{k}-1$ and $\hat{k}-1\geq k$, by Lemma 4, vector $\left(\ldots ,|{\dot{X}}_{c_{a}}^{\hat{k}-1}|-1,|{\dot{X}}_{c_{j}}^{\hat{k}-1}|+1,\ldots \right)$ is feasible. Recall that school *c*_*j*_’s student number does not increase strictly between *k* and *k*′. Moreover, since $|{\dot{X}}_{c_{a}}^{\hat{k}-1}\left|=\right|{\dot{X}}_{c_{j}}^{k}\left|+2\geq \right|{\dot{X}}_{c_{j}}^{\hat{k}-1}|+2$, school *c*_*j*_ cannot be full at stage $\hat{k}$, which implies that school *c*_*j*_’s student number does not decrease between *k* and $\hat{k}$. Therefore, we obtain $|{\dot{X}}_{c_{j}}^{\hat{k}-1}\left|=\right|{\dot{X}}_{c_{j}}^{k}|$. Since $|{\dot{X}}_{c_{b}}^{\hat{k}-1}\left|<\right|{\dot{X}}_{c_{j}}^{k}|+1$, it leads to $|{\dot{X}}_{c_{b}}^{\hat{k}-1}\left|<\right|{\dot{X}}_{c_{j}}^{\hat{k}-1}|+1$. Thus, by Lemma 1 on vector $\left(\ldots ,|{\dot{X}}_{c_{a}}^{\hat{k}-1}|-1,|{\dot{X}}_{c_{j}}^{\hat{k}-1}|+1,\ldots \right)$, vector $\left(\ldots ,|{\dot{X}}_{c_{a}}^{\hat{k}-1}|-1,|{\dot{X}}_{c_{b}}^{\hat{k}-1}|+1,\ldots \right)$ is also feasible, i.e., vector $\zeta ({\dot{X}}^{\hat{k}})$ is feasible. Hence, QRDA terminates strictly before *k*′, which is a contradiction.

Last, for case (ii), we can create a new scenario, $C_{s}^{\prime \prime }$, by removing all the schools that are less desired than *c*_*j*_ based on ≻_*s*_ from $C_{s}^{\prime }$. If *s* is assigned to *c*_*j*_ in $R(C_{s}^{\prime \prime })$, we obtain the same contradiction as for case (i) by comparing $R(C_{s}^{\prime \prime })$ and $R(C_{s})$. Thus, action “school *c*_*j*_ rejects student *s*” must appear in $R(C_{s}^{\prime \prime })$. By Lemma 3, this action also appears in $R(C_{s}^{\prime })$, but this also leads to a contradiction.

QRDA’s strategyproofness is heavily based on the fact that *σ* is balanced. Under ratio constraints, Yahiro et al. [21] provided an example to show that QRDA is not strategyproof if *σ* is not balanced. To be self-contained, we reproduce this example with Example 7.

**Example 7**. *Consider S* = {*s*_1_, …, *s*_9_}, *C* = {*c*_1_, *c*_2_, *c*_3_}, *α* = 1/4 *(the ratio constraints), and σ starting with c*_2_, *c*_2_, *c*_1_. *The following are the preferences of the students and the schools*:

$$\begin{array}{c} \;s_{1},\ldots ,\;s_{5}\;:\;c_{1}≻c_{2}≻c_{3}\;\\ s_{6},s_{7},s_{8}\;:\;c_{2}≻c_{3}≻c_{1}\;\\ s_{9}\;:\;c_{3}≻c_{1}≻c_{2}\; \end{array}c_{1},\;c_{2},\;c_{3}\;:\;s_{1}≻s_{2}≻s_{3}≻s_{4}≻s_{6}≻s_{7}≻s_{8}≻s_{9}≻s_{5}.$$

*First, QRDA*^*σ*^ *sets* $q_{c}^{1}=5$ , *for all c* ∈ *C*. *At stage* 1, *all students are assigned to their favorite schools, but this is not feasible. At both stages* 2 *and* 3, *the quota of c*_2_ *is decreased by one, although the matching remains the same. Then at stage 4, the quota of c*_1_ *is decreased by one and s*_5_ *is rejected from c*_1_. *Student s*_5_ *applies to c*_2_, *which also rejects her. She is finally assigned to c*_3_ *and the corresponding matching is feasible. However, if student s*_5_ *misreports by declaring that c*_2_ *is her most preferred school, QRDA stops at stage 1 by returning a feasible matching where she is assigned to school c*_2_. *Hence, student s*_5_ *manipulated the mechanism to get a better result (s*_5_, *c*_2_).

Under ratio constraints, QRDA has been proven to hold additional gratifying properties [21], most of which can be trivially extended to our setting. Their proofs for weak non-bossiness, weak Maskin monotonicity, weak group strategyproofness, and non-domination rely only on QRDA’s strategyproofness under ratio constraints and DA’s properties. Since Theorem 2 shows that QRDA is strategyproof in our setting, Theorems 3 and 4 trivially hold.

**Theorem 3**. *QRDA is weakly non-bossy, weakly Maskin monotone, and weakly group strategyproof*.

**Theorem 4**. *Given a balanced σ, no strategyproof mechanism exists that dominates QRDA*^*σ*^.

However, weak Pareto optimality does not trivially extend to a union of symmetric M-convex sets since its proof under ratio constraints strongly relies on the structure of the ratio constraints. With Theorem 5, we show that QRDA remains weakly Pareto optimal in our setting.

**Theorem 5**. *QRDA is weakly Pareto optimal*.

*Proof*. Toward a contradiction, assume a matching $\dot{X}$ that strongly dominates the matching returned by QRDA, denoted by $\ddot{X}$, i.e., ${\dot{X}}_{s}{≻}_{s}{\ddot{X}}_{s}$ holds for all *s* ∈ *S*. Let *k* denote the last stage of QRDA, i.e., ${\ddot{X}}^{k}=\ddot{X}$. The proof is conducted by showing that matching ${\ddot{X}}^{k-1}$ is feasible, i.e., the matching returned by QRDA at stage *k* − 1 is feasible, which contradicts that QRDA terminates at stage *k*. We develop the proof through arguments (A), (B), and (C).

(A) This argument shows that for all *c* ∈ *C* such that $|{\ddot{X}}_{c}^{k}|=0$, it holds $|{\dot{X}}_{c}|=0$.

First, we show that for all *c* ∈ *C*, $q_{c}^{k}\geq 1$. Assume *c*′ ∈ *C* exists such that $q_{c^{\prime }}^{k}=0$. The definition of QRDA’s quota reduction sequence implies that for all *c* ∈ *C*, both $q_{c}^{k}\leq 1$ and $q_{c}^{k-1}\leq 1$ hold. Therefore, for any school *c* ∈ *C*, both $|{\ddot{X}}_{c}^{k}|\leq 1$ and $|{\ddot{X}}_{c}^{k-1}|\leq 1$ hold. Hence, vector $\zeta ({\ddot{X}}^{k-1})$ is a permutation of vector $\zeta ({\ddot{X}}^{k})$ and thus, by symmetry, ${\ddot{X}}^{k-1}$ is feasible, which contradicts that QRDA terminates at stage *k*.

Then consider a school *c*′ ∈ *C* such that $|{\ddot{X}}_{c^{\prime }}^{k}|=0$. Since for all *c* ∈ *C*, $q_{c}^{k}\geq 1$, no student applies to *c*′ during QRDA, implying that for all *s* ∈ *S*, ${\ddot{X}}_{s}^{k}{≻}_{s}\;c^{\prime }$. If $|{\dot{X}}_{c^{\prime }}|\neq 0$, then for any student *s* assigned to *c*′ in $\dot{X}$, it holds that $c^{\prime }{≻}_{s}\;{\ddot{X}}_{s}^{k}$, which is a contradiction.

(B) The next argument shows that any school *c* such that $|{\dot{X}}_{c}|\neq 0$ is full at stage *k* − 1 of QRDA, i.e., $|{\ddot{X}}_{c}^{k-1}|=q_{c}^{k-1}$.

Consider a school *c*′, which is not full at stage *k* − 1, implying that it rejects no student during QRDA. Toward a contradiction, assume that $|{\dot{X}}_{c^{\prime }}|\neq 0$, and consider a student *s*′ who is assigned to *c*′ in $\dot{X}$. Since $\dot{X}$ strongly dominates $\ddot{X}$, ${\dot{X}}_{s^{\prime }}{≻}_{s^{\prime }}{\ddot{X}}_{s^{\prime }}$ holds. Hence, during QRDA, student *s*′ has to be rejected by school *c*′ before being allowed to apply to ${\ddot{X}}_{s^{\prime }}$, which contradicts that *c*′ rejects no student during QRDA.

(C) Finally, we show that we can transform matching $\dot{X}$ into matching ${\ddot{X}}^{k-1}$ by applying permutations and operations that satisfy the conditions of Lemma 1.

Denote $C^{\prime }=\{c\in C||{\dot{X}}_{c}\left|\neq 0\right\}$ and thus $\sum_{c\in C^{\prime }}\left|{\dot{X}}_{c}\right|=n$. Let matching $\tilde{X}$ be one of the matchings such that (i) for all *c* ∈ *C*\*C*′, $|{\tilde{X}}_{c}|=0$, and (ii) for all *c* ∈ *C*′, $|{\tilde{X}}_{c}\left|=\lceil n/\right|C^{\prime }\left|\rceil \;\text{or}\;\lfloor n/\right|C^{\prime }\left|\right\rfloor$, i.e., $\tilde{X}$ is one of the most balanced matchings when we restrict the setting to schools in *C*′. With a similar argument as proof of Lemma 2, we can show that any matching can be transformed into a most balanced matching only by permutations and operations satisfying the conditions of Lemma 1. Hence, since $\tilde{X}$ is one of the most balanced vectors when we restrict schools to *C*′, we can transform matching $\dot{X}$ into $\tilde{X}$ only by permutations and operations satisfying the conditions of Lemma 1. Therefore, since $\dot{X}$ is feasible when considering all schools *C*, $\tilde{X}$ is also feasible when considering all schools *C*.

By definition of $\tilde{X}$, $\sum_{c\in C^{\prime }}\left|{\tilde{X}}_{c}\right|=n$ holds, and thus $\sum_{c\in C^{\prime }}|{\tilde{X}}_{c}|\geq \sum_{c\in C^{\prime }}\left|{\ddot{X}}_{c}^{k-1}\right|$ also holds. Furthermore, by argument (B), all schools *c* ∈ *C*′ are full at stage *k* − 1, i.e., $|{\ddot{X}}_{c}^{k-1}|=q_{c}^{k-1}$. With QRDA’s quota reduction sequence, it implies that for all *c* ∈ *C*, $q_{c}^{k-1}\leq \left\lceil n/\right|C^{\prime }\left|\right\rceil$. Therefore, in addition to satisfying (i) and (ii), matching $\tilde{X}$ can be chosen such that it also satisfies (iii) for all *c* ∈ *C*′, $|{\tilde{X}}_{c}\left|\geq \right|{\ddot{X}}_{c}^{k-1}|$. Now note that $\sum_{c\in C^{\prime }}|{\tilde{X}}_{c}|=n=\sum_{c\in C}\left|{\ddot{X}}_{c}^{k-1}\right|$ holds, and, with condition (iii), $\sum_{c\in C^{\prime }}\left(\right|{\tilde{X}}_{c}\left|-\right|{\ddot{X}}_{c}^{k-1}\left|\right)=\sum_{c\in C\backslash C^{\prime }}\left|{\ddot{X}}_{c}^{k-1}\right|$ holds. It intuitively means that the surplus of students in the schools of *C*′ when comparing $\tilde{X}$ and ${\ddot{X}}^{k-1}$ equals the student number in the schools of *C*\*C*′ in ${\ddot{X}}^{k-1}$. To transform $\tilde{X}$ into ${\ddot{X}}^{k-1}$, we can transfer the surplus of students from a school in *C*′ to a school in *C*\*C*′ with operations that satisfy the conditions of Lemma 1 and with permutations.

${\ddot{X}}^{k-1}$ is feasible by Lemma 1 and symmetry, which is a contradiction. Hence, no matching exists that strongly dominates $\ddot{X}$.

As Proposition 2 shows, fairness and nonwastefulness are incompatible. Since QRDA is fair, it cannot be nonwasteful. Nevertheless, QRDA satisfies the following weaker version of nonwastefulness.

**Definition 18** (Weak Nonwastefulness). *In matching* $\dot{X}$, *where* $\left(s,c\right)\in \dot{X}$, *student s strongly claims an empty seat in c*′ *if* (*s*, *c*′) ≻_*s*_ (*s*, *c*), $\left(\dot{X}\backslash \left\{\left(s,c\right)\right\}\right)\cup \left\{\left(s,c^{\prime }\right)\right\}$ *is school-feasible, and* $|{\dot{X}}_{c^{\prime }}\left|+2\leq \right|{\dot{X}}_{c}|$. Matching $\dot{X}$ *is weakly nonwasteful if no student strongly claims an empty seat in* $\dot{X}$. *A mechanism is weakly nonwasteful if it always outputs a weakly nonwasteful matching*.

Intuitively, a student strongly claims an empty seat if, by unilaterally moving the student to her more preferred school, the result allocation vector is not only school-feasible but also more balanced than the original allocation vector; if we move *s* from school *c* to school *c*′, the student number in *c*′ is still fewer than or equal to that in *c*.

We have the following result in terms of this weaker version of nonwastefulness.

**Theorem 6**. *QRDA is weakly nonwasteful when* V *is a union of symmetric M-convex sets*.

*Proof*. We prove this theorem towards a contradiction. Assume that QRDA terminates at stage *k*, outputting feasible matching ${\dot{X}}^{k}$. Student *s* exists such that $\left(s,c\right)\in {\dot{X}}^{k}$ and *s* strongly claims an empty seat in school *c*′ in matching ${\dot{X}}^{k}$. According to Definition 18, *s* prefers *c*′ over *c* and $|{\dot{X}}_{c^{\prime }}^{k}\left|+2\leq \right|{\dot{X}}_{c}^{k}|$ holds. Since *s* prefers *c*′ over *c*, *s* must have applied to *c*′ and been rejected. Thus, *c*′ must be full, i.e., $|{\dot{X}}_{c^{\prime }}^{k}|=q_{c^{\prime }}^{k}$ holds. Also, $|{\dot{X}}_{c}^{k}|\leq q_{c}^{k}$ must hold, since school *c*’s student number cannot exceed its quota. By the fact that $|{\dot{X}}_{c^{\prime }}^{k}\left|+2\leq \right|{\dot{X}}_{c}^{k}|$ holds, we obtain $q_{c^{\prime }}^{k}+2\leq q_{c}^{k}$. However, this contradicts the fact that the quota reduction sequence is balanced, that is, the difference between the maximum quotas of any pair of schools is at most one.

Lastly, we briefly delve into the time complexity of QRDA. At each stage *k* of QRDA, we do not have to run DA from the beginning (that is, each student starts by applying to the first-ranked school). Instead, at stage *k* of QRDA, each student first applies to her most preferred school among those that have not rejected her at all previous stages. This modification ensures that the resulting matching remains unaffected, as the execution order in DA can be flexible [40].

**Theorem 7**. *The time complexity of QRDA is O*(*mn*), *assuming that verifying the school-feasibility of a vector can be done in constant time*.

QRDA repeatedly applies the standard DA. By the above modification, a student is rejected by each school at most once during the whole QRDA’s execution. Thus, each step in DA is executed at most *mn* times in total. Hence, the time complexity of QRDA is *O*(*mn*).

### 4.3 Theoretical comparison with baseline mechanism

To the best of our knowledge, no fair and strategyproof mechanism exists in the literature that can handle the union of symmetric M-convex constraints except QRDA. An alternative approach to address such distributional constraints is by transforming them into artificial maximum quotas, thereby ensuring that standard DA returns a matching that satisfies these constraints. This method, called Artificial Cap Deferred Acceptance (ACDA), inherits DA’s properties while losing some of the flexibility of the original constraints due to the restriction of the set of feasible matchings. ACDA is employed in Japanese medical resident matching programs [2] and serves as a benchmark mechanism in various works focused on distributional constraints [4, 5, 30].

If prior information were available regarding the popularity of schools, it might be possible to set *q*_*C*_ to meet distributional constraints and maximize student welfare. Otherwise, a pragmatic and rational approach to determine suitable *q*_*C*_ is by utilizing a most balanced vector. Similar to QRDA, ACDA is defined with respect to a specific sequence *σ*, denoted as ACDA^*σ*^. We assume that *σ* is the round-robin order *c*_1_, *c*_2_, …, *c*_*m*_, unless otherwise specified. ACDA is defined as follows:

**Mechanism 3** (ACDA (based on a most balanced vector))

**Initialization:**
  *For each i where* i≤(nmodm) , $q_{c_{i}}\leftarrow \left\lfloor n/m\right\rfloor$, and for each *i* where i>(nmodm), $q_{c_{i}}\leftarrow \left\lceil n/m\right\rceil$.
**Execution:**
  *Run standard DA in market* (*S*,*C*,≻_*S*_,≻_*C*_,*q*_*C*_).

As stated above, ACDA inherits the properties of DA like fairness and strategyproofness.

**Theorem 8**. *ACDA is strategyproof and returns a feasible and fair matching*.

*Proof*. Since all the most balanced vectors are included in V, the matching obtained under *q*_*C*_ is clearly feasible. Since standard DA is fair [18], the matching obtained by ACDA is also fair. Furthermore, since *q*_*C*_ is determined independently from ≻_*S*_ and the standard DA is strategyproof, ACDA is strategyproof.

The next result shows that QRDA is overwhelmingly favored by stating that all students weakly prefer the school that they obtain in QRDA to the one in ACDA.

**Theorem 9**. *Given a balanced sequence σ*, *all students weakly prefer the matching obtained by QRDA*^*σ*^ *over that by ACDA*^*σ*^.

*Proof*. Let *k*′ denote the stage of QRDA^*σ*^ where $q_{C}^{k^{\prime }}$ becomes identical to the *q*_*C*_ used in ACDA^*σ*^. This situation can happen since ACDA^*σ*^ generates the most balanced vector based on the quota reduction sequence *σ*. As shown in the proof of Theorem 1, QRDA^*σ*^ terminates at stage *k* where *k* ≤ *k*′. Since $q_{c}^{k}\geq q_{c}^{k^{\prime }}$ holds for all *c* ∈ *C* and DA satisfies resource monotonicity (described in the proof of Theorem 2), each student weakly prefers the matching obtained by QRDA^*σ*^ over that by ACDA^*σ*^.

Since QRDA always obtains a (weakly) better matching for students than ACDA, one may assume that QRDA is always less wasteful than ACDA, i.e., more students claim empty seats in ACDA compared to QRDA. However, we cannot guarantee that QRDA is less wasteful than ACDA. Yahiro et al. [21] identified a case where the number of students who claim empty seats in QRDA exceeds that of ACDA under ratio constraints.

**Theorem 10** (based on Theorem 11 by Yahiro et al. [21]). *Given a balanced σ*, *a case exists where the number of students who claim empty seats in QRDA*^*σ*^ *exceeds that of ACDA*^*σ*^.

Theorem 10 implies that there are cases where QRDA still has room for amelioration by mitigating students’ claims compared to ACDA. Under ratio constraints, this QRDA’s drawback is compensated by the fact that when ACDA returns a nonwasteful matching, QRDA returns the same nonwasteful matching [21]. Theorem 11 shows that this fact extends to a union of symmetric M-convex sets.

**Theorem 11**. *Given a balanced σ*, *when ACDA*^*σ*^ *returns a nonwasteful matching, QRDA*^*σ*^ *and ACDA*^*σ*^ *return the same matching*.

*Proof*. In market $\left(S,C,{≻}_{S},{≻}_{C},V\right)$, assume that QRDA^*σ*^ returns matching $\ddot{X}$ and that ACDA^*σ*^ returns matching $\dot{X}$ that differs from $\ddot{X}$. By contradiction assume also that $\dot{X}$ is nonwasteful.

Consider a procedure that starts with matching $\ddot{X}$ and keeps applying QRDA’s stages, i.e., reducing quotas and applying DA (even though $\ddot{X}$ is feasible) until the quotas reach the same quotas as in ACDA^*σ*^, and thus the procedure returns $\dot{X}$. During this procedure, since $\dot{X}$ differs from $\ddot{X}$, some rejection chains must occur and let *r* denote the last of these rejection chains. Assume rejection chain *r* decreases the student number in school *c*′ and increases *c*′′. Denote by ${\dot{X}}^{-r}$ the matching returned by this procedure before rejection chain *r* happens, i.e., $\zeta \left({\dot{X}}^{-r}\right)=\left(\right|{\dot{X}}_{c_{1}}\left|,\ldots ,\right|{\dot{X}}_{c^{\prime }}\left|+1,\right|{\dot{X}}_{c^{\prime \prime }}\left|-1,\ldots ,\right|{\dot{X}}_{c_{m}}\left|\right)$. Since $\ddot{X}$ is feasible, by Lemma 1, any matching returned during this procedure is feasible, in particular ${\dot{X}}^{-r}$ is feasible. In the following, we show that one student involved in the rejection chain *r* is a claiming student in $\dot{X}$.

Notice that each school that rejects a student during rejection chain *r* has to be full in matching ${\dot{X}}^{-r}$, and can be either maximum or not in ${\dot{X}}^{-r}$, i.e., its student number can be $|{\dot{X}}_{c^{\prime }}^{-r}|$ or $|{\dot{X}}_{c^{\prime }}^{-r}|-1$.

First, we assume that rejection chain *r* involves a rejection from a school that is maximum in ${\dot{X}}^{-r}$. Consider the first maximum school in ${\dot{X}}^{-r}$ that rejects a student in *r*, denoted as *c*_*b*_, and then $|{\dot{X}}_{c_{b}}^{-r}\left|=\right|{\dot{X}}_{c^{\prime }}^{-r}|$. Assume that school *c*_*b*_ accepts student *s*_*b*_ (and then rejects another one), and that *s*_*b*_ was initially rejected by school *c*_*a*_. We have two cases to consider: either *c*_*a*_ = *c*′ or *c*_*a*_ ≠ *c*′.

If *c*_*a*_ = *c*′, consider matching $\left(\dot{X}\backslash \left\{\left(s_{b},c_{b}\right)\right\}\right)\cup \left\{\left(s_{b},c^{\prime }\right)\right\}$. Then note that $\zeta \left(\left(\dot{X}\backslash \left\{\left(s_{b},c_{b}\right)\right\}\right)\cup \left\{\left(s_{b},c^{\prime }\right)\right\}\right)=\left(\right|{\dot{X}}_{c_{1}}\left|,\ldots ,\right|{\dot{X}}_{c^{\prime }}\left|+1,\right|{\dot{X}}_{c_{b}}\left|-1,\ldots ,\right|{\dot{X}}_{c_{m}}\left|\right)$. By assumption, $|{\dot{X}}_{c_{b}}^{-r}\left|=\right|{\dot{X}}_{c^{\prime }}^{-r}|$, and since $|{\dot{X}}_{c^{\prime }}^{-r}\left|=\right|{\dot{X}}_{c^{\prime }}|+1$ and $|{\dot{X}}_{c_{b}}^{-r}\left|=\right|{\dot{X}}_{c_{b}}|$, vector $\zeta (\left(\dot{X}\backslash \left\{\left(s_{b},c_{b}\right)\right\}\right)\cup \left\{\left(s_{b},c^{\prime }\right)\right\})$ is a permutation of $\dot{X}$. By symmetry, matching $\left(\dot{X}\backslash \left\{\left(s^{\prime },c_{b}\right)\right\}\right)\cup \left\{\left(s^{\prime },c^{\prime }\right)\right\}$ is feasible, and thus student *s*_*b*_ is a claiming student in $\dot{X}$.

Otherwise, *c*_*a*_ ≠ *c*′, and consider matching $\left(\dot{X}\backslash \left\{\left(s_{b},c_{b}\right)\right\}\right)\cup \left\{\left(s_{b},c_{a}\right)\right\}$. Note that $\zeta \left(\left(\dot{X}\backslash \left\{\left(s_{b},c_{b}\right)\right\}\right)\cup \left\{\left(s_{b},c_{a}\right)\right\}\right)=\left(\right|{\dot{X}}_{c_{1}}\left|,\ldots ,\right|{\dot{X}}_{c_{a}}\left|+1,\right|{\dot{X}}_{c_{b}}\left|-1,\ldots ,\right|{\dot{X}}_{c_{m}}\left|\right)$. By definition of *c*_*b*_, school *c*_*a*_ is not maximum in ${\dot{X}}^{-r}$, and thus $|{\dot{X}}_{c_{a}}^{-r}\left|=\right|{\dot{X}}_{c^{\prime }}^{-r}|-1$ holds. In addition with $|{\dot{X}}_{c_{b}}^{-r}\left|=\right|{\dot{X}}_{c_{b}}|$, it implies that vector $\zeta (\left(\dot{X}\backslash \left\{\left(s_{b},c_{b}\right)\right\}\right)\cup \left\{\left(s_{b},c_{a}\right)\right\})$ is a permutation of $\dot{X}$. By symmetry, matching $\left(\dot{X}\backslash \left\{\left(s_{b},c_{b}\right)\right\}\right)\cup \left\{\left(s_{b},c_{a}\right)\right\}$ is feasible, and thus student *s*_*b*_ is a claiming student in $\dot{X}$.

Therefore, assume no rejection exists in *r* that involves a school that is maximum in ${\dot{X}}^{-r}$. Consider the *last* rejection in rejection chain *r*, and assume that school *c*_*d*_ rejects student *s*_*d*_ who is then accepted by school *c*′′ (and rejection chain *r* stops). Note that $\zeta \left(\left(\dot{X}\backslash \left\{\left(s_{d},c^{\prime \prime }\right)\right\}\right)\cup \left\{\left(s_{d},c_{d}\right)\right\}\right)=\left(\right|{\dot{X}}_{c_{1}}\left|,\ldots ,\right|{\dot{X}}_{c_{d}}\left|+1,\right|{\dot{X}}_{c^{\prime \prime }}\left|-1,\ldots ,\right|{\dot{X}}_{c_{m}}\left|\right)$. By assumption, since school *c*_*d*_ is not maximum in ${\dot{X}}^{-r}$, $|{\dot{X}}_{c_{d}}^{-r}\left|=\right|{\dot{X}}_{c^{\prime }}^{-r}|-1$. Then vector $\zeta (\left(\dot{X}\backslash \left\{\left(s_{d},c^{\prime \prime }\right)\right\}\right)\cup \left\{\left(s_{d},c_{d}\right)\right\})$ is a permutation of ${\dot{X}}^{-r}$. By symmetry, matching $\left(\dot{X}\backslash \left\{\left(s_{d},c^{\prime \prime }\right)\right\}\right)\cup \left\{\left(s_{d},c_{d}\right)\right\}$ is feasible, and thus student *s*_*d*_ is a claiming student in $\dot{X}$.

## 5 Generalized asymmetric constraints: Union of symmetric M-convex sets with an offset

In Section 3.3, we introduced the union of symmetric M-convex constraints, each component of which is symmetric. Symmetry assumes that all schools have approximately equal size, which might not hold true in real-world scenarios where schools have different sizes and enrollment capacities. To address such cases, we extend the symmetric M-convex constraints by introducing an asymmetric factor called an *offset*.

### 5.1 Union of symmetric M-convex constraints with an offset

A union of symmetric M-convex constraints with an offset is defined as follows.

**Definition 19** (Union of Symmetric M-convex Sets with an Offset). *Set* $V\subseteq N_{0}^{m}$ *is a union of symmetric M-convex sets with an offset if offset vector* $\nu^{*}\in Z^{m}$ *and* $V^{\prime }\subseteq N_{0}^{m}$ *exist such that* $V^{\prime }$ *is a union of symmetric M-convex sets and* V *is given as* $\left\{\nu |\nu^{\prime }\in V^{\prime },\nu =\nu^{\prime }+\nu^{*},\sum_{c\in C}\nu_{c}=n\right\}$.

In short, a union of symmetric M-convex sets with an offset is derived from a union of symmetric M-convex sets $V^{\prime }$ by adding offset vector ν* to each element in $V^{\prime }$. Note that offset vector ν* may contain negative elements, as long as each element in ν∈V is non-negative.

One practical example of a union of symmetric M-convex sets with an offset is when each school requires a specific number of students to operate, represented as its minimum quota. More specifically, each school *c* has its own minimum quota *p*_*c*_, which is defined analogously to Definition 11. However, these minimum quotas may vary based on the size of the schools. We apply the union of symmetric M-convex constraints to the number of surplus students beyond *p*_*c*_ for all *c* ∈ *C*, rather than the number of overall students. The market is denoted by a tuple $\left(S,C,{≻}_{S},{≻}_{C},p_{C},V\right)$. In this market, the constraints are represented as follows.

1. The individual minimum quotas, $p_{C}=\left(p_{c_{1}},\ldots ,p_{c_{m}}\right)$, which is an *m*-dimensional vector and each *p*_*c*_ is the minimum quota of school *c*. We require ∑_*c*∈*C*_ *p*_*c*_ < *n*.
2. The constraints on the number of surplus students for each school beyond *p*_*C*_ are given as a union of *m*-dimensional symmetric M-convex sets $V\subseteq N_{0}^{m}$.
3. Matching $\dot{X}$ is feasible if $\zeta \left(\dot{X}\right)-p_{C}\in V$ holds.

### 5.2 QRDA for union of symmetric M-convex constraints with an offset

To handle this generalized class of constraints, we slightly modify our proposed mechanism, QRDA, with different initial individual maximum quotas to handle the asymmetry introduced by the offset vector. Recall that under the constraints in Section 3.3, QRDA commences with uniform maximum quota *ν*^*max*^, which satisfies $\nu^{max}\geq {\text{max}}_{\nu \in V,i\in [m]}\nu_{i}$ for each school, and reduces the schools’ quotas based on a given sequence *σ*. On the other hand, for any union of symmetric M-convex sets with an offset V, we define ${\tilde{\nu }}^{max}$ as a value that satisfies ${\tilde{\nu }}^{max}\geq {\text{max}}_{\nu^{\prime }\in V^{\prime },i\in \left[m\right]}\nu_{i}^{\prime }$. For each school *c*, $\nu_{c}^{max}$ is defined as ${\tilde{\nu }}^{max}+\nu_{c}^{*}$. In short, $\nu_{c}^{max}$ is the sum of the common upper-bound ${\tilde{\nu }}^{max}$ and the offset of *c*. The modified QRDA sets $q_{c}^{1}$ to $\nu_{c}^{max}$, signifying that the initial maximum quota for each school is an upper-bound for any possible value in V. Intuitively, in the generalized constraints handled in this section, a set of “most balanced vectors” still exists, although they have been shifted by the offset vector. The constraints are therefore symmetric and M-convex around the most balanced vectors. Therefore, the modified QRDA ensures that any returned matching still satisfies all the properties described above. Applying similar arguments in the previous sections, we deduce the following theorem.

**Theorem 12**. *In any market under a union of symmetric M-convex constraints with an offset, the modified QRDA is feasible, fair, and strategyproof*.

## 6 Experimental evaluation

Concerning student welfare, Theorem 9 guarantees that students weakly prefer QRDA over ACDA. To delve deeper into the quantitative disparities in student welfare between QRDA and ACDA, we rely on computer simulations for a comprehensive analysis. On the other hand, Theorem 10 shows that we cannot guarantee that QRDA is always better than ACDA in terms of nonwastefulness. Neverthless, our conjecture remains that situations in which QRDA is more wasteful than ACDA are likely to be rare and atypical; in the majority of cases, QRDA should prove superior. This conjecture is substantiated by our computer simulations. We undertake extensive investigations into these inquiries concerning both difference constraints and flexible uniform min/max quotas constraints. Additionally, for difference constraints, we explore an alternative indirect approach, whereby the union of symmetric M-convex constraints is transformed into individual min/max quotas constraints. Within this method, each school *c* has its minimum quota *p*_*c*_ as well as its maximum quota *q*_*c*_. At least *p*_*c*_ and at most *q*_*c*_ students have to be assigned to school *c*. Fragiadakis et al. [4] present a strategyproof and fair mechanism called Extended Seat Deferred Acceptance (ESDA) to handle individual min/max quotas constraints. As previously mentioned in Section 3.3.1, difference constraints with parameter *β* can be represented by a union of uniform min/max quotas constraints. To adapt ESDA to handle difference constraints, we calculate uniform min/max quotas $\tilde{p}$ and $\tilde{q}$: choose the maximum value of minimum quota $\tilde{p}$, with corresponding maximum quota $\tilde{q}=\tilde{p}+\beta$, such that $\tilde{p}$ and $\tilde{q}$ satisfy $\tilde{q}+\left(m-1\right)\tilde{p}\leq n\leq \left(m-1\right)\tilde{q}+\tilde{p}$. In simpler terms, we choose the largest value of $\tilde{p}$ among the union of uniform min/max quotas constraints. The minimum quota $\tilde{p}$ signifies the minimum number of students to be allocated to each school, while the maximum quota $\tilde{q}$ allows for more students to attend popular schools. We implemented alternative methods for choosing $\tilde{p}$ and $\tilde{q}$, such as selecting the smallest $\tilde{p}$ from the union of uniform min/max quotas constraints. Remarkably, the results obtained from these alternative methods were similar.

We consider a market with *n* = 800 students and *m* = 20 schools. We generate student preferences using the Mallows model [43, 44]. The strict preference ≻_*s*_ of each student *s* probabilistically determined, with the probability expressed as follows:

$$\begin{array}{c} \text{Pr}\left({≻}_{s}\right)=\frac{\text{exp}(-\theta \cdot \omega \left({≻}_{s},{≻}_{\hat{s}}\right))}{\sum_{{≻}_{s}^{\prime }}\text{exp}\left(-\theta \cdot \omega \left({≻}_{s}^{\prime },{≻}_{\hat{s}}\right)\right)}. \end{array}$$

In this equation, θ∈R denotes a spread parameter, ${≻}_{\hat{s}}$ is a central preference (uniformly randomly chosen from all possible preferences in our experiment), and $\omega ({≻}_{s},{≻}_{\hat{s}})$ represents the Kendall tau distance, which counts the number of pairwise inversions between ≻_*s*_ and ${≻}_{\hat{s}}$. When *θ* = 0, the Mallows model reduces to the uniform distribution, equivalent to the impartial culture in our setting. As *θ* increases, all student preferences tend to converge toward the constant distribution that returns ${≻}_{\hat{s}}$. To ensure reliable findings, we choose two values for *θ*, 0.1 and 0.3, reflecting real-world diversity in preferences and some schools being more preferred. The priority ranking of each school *c* is drawn uniformly at random. We create 100 problem instances for each parameter setting.

We first conduct a computer simulation with *difference constraints* (Definition 16) to quantitatively measure the weak domination of QRDA over ACDA and to show that QRDA outperforms ACDA in terms of nonwastefulness.

**Fig 2:**
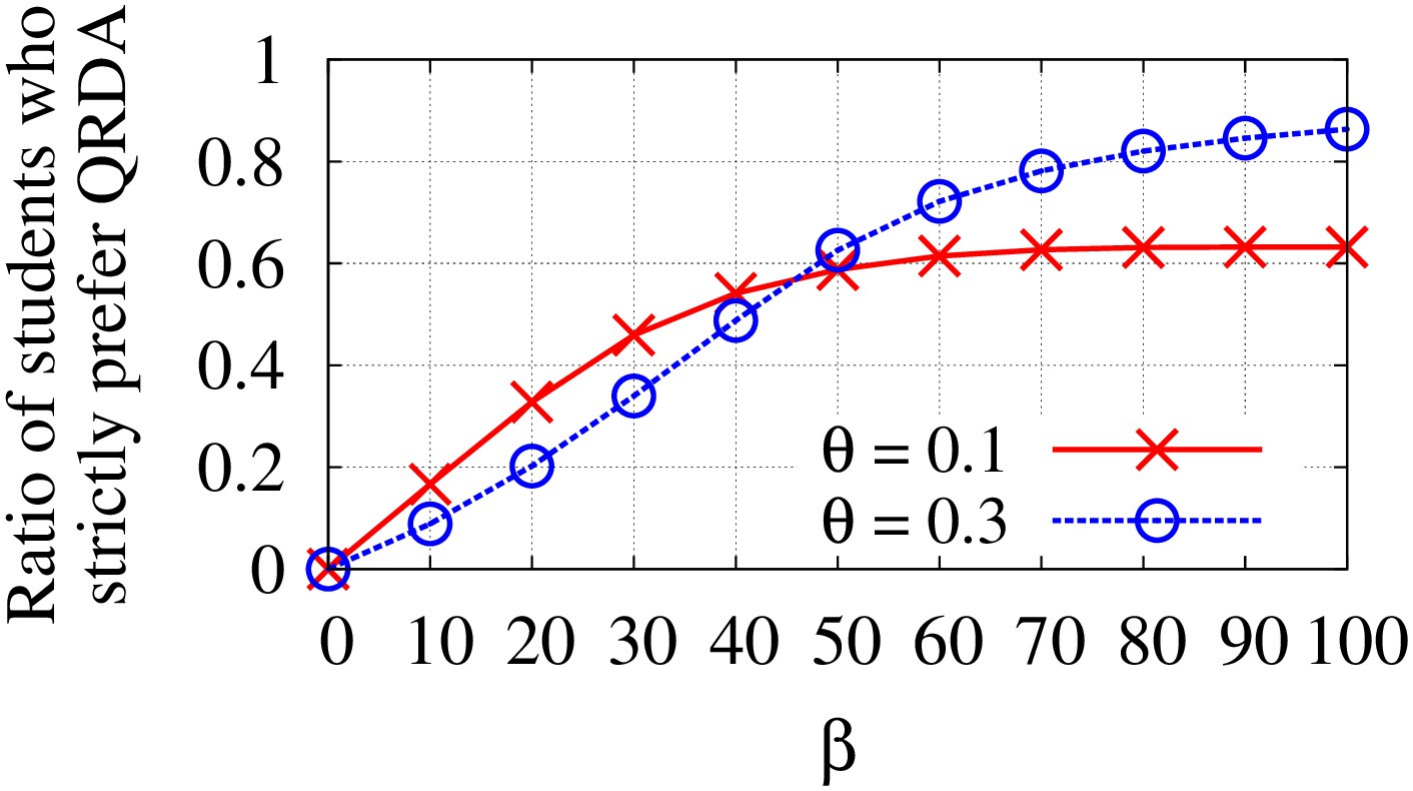
Comparisons between QRDA and ACDA under difference constraints: Ratio of students who strictly prefer QRDA to ACDA. ⊖ denotes the values when *θ* = 0.1 and × denotes the values when *θ* = 0.3.

In Fig 2, we show the proportion of students who strictly prefer QRDA over ACDA depending on the allowed difference *β* in Definition 16. When *β* = 10 and *θ* = 0.1, approximately 18% of the students strictly prefer QRDA’s outcome; this number increases before plateauing at 60% when *β* = 50. As *β* increases, since the set of feasible sets expands, the potential of amelioration with QRDA increases as well. We expect policymakers to prefer QRDA over ACDA since it outperforms ACDA and a significant amount of students strictly prefer it. When *θ* = 0.3, the competition among students rises since their preferences tend to be more similar. Compared to *θ* = 0.1, the improvement by QRDA is smaller when *β* is small (*β* ≤ 45) and larger when *β* ≥ 45. A reason might be that as the competition becomes more severe (*θ* = 0.3), it is more difficult to enhance students’ welfare since the resources in popular schools are limited even though QRDA has more flexibility.

**Fig 3:**
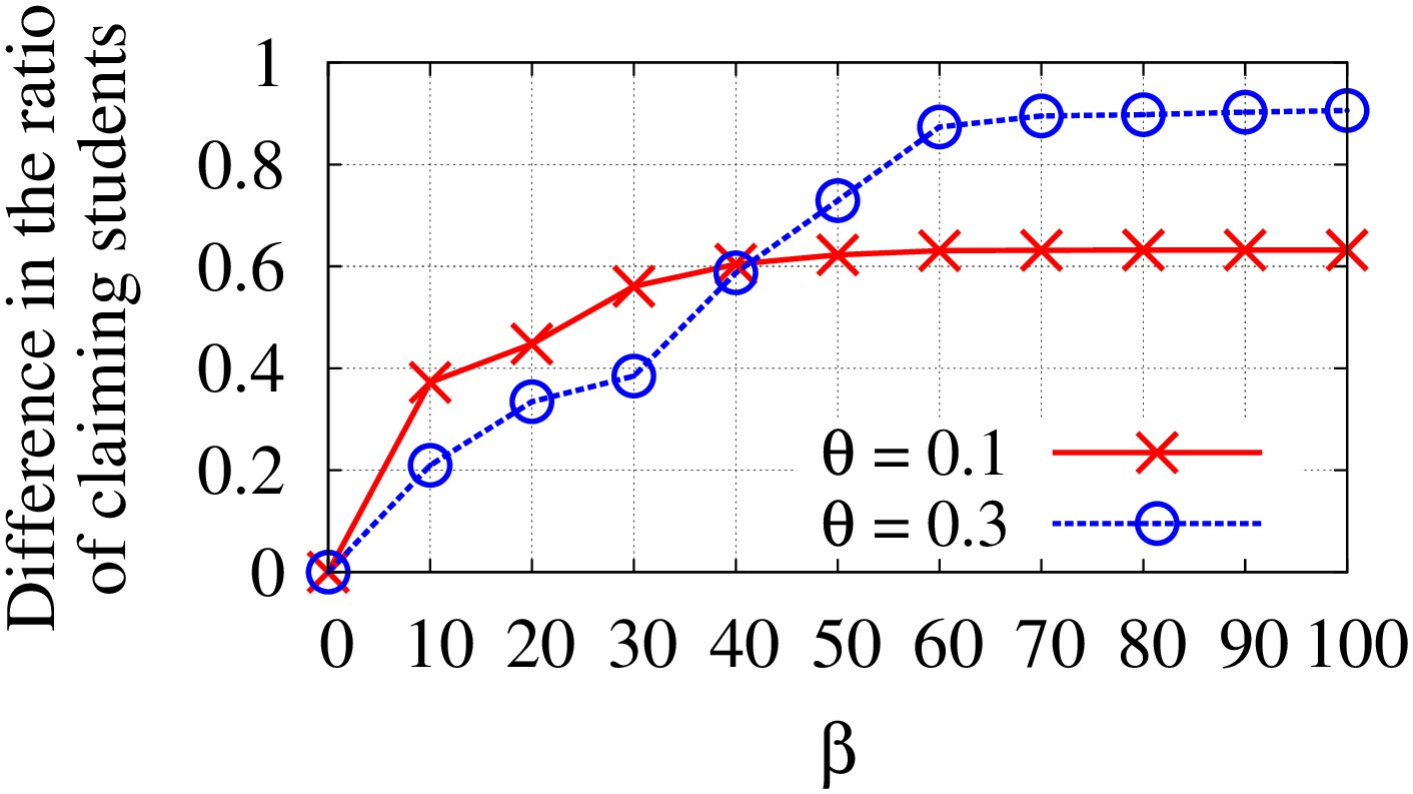
Comparisons between QRDA and ACDA under difference constraints: Difference between ratio of claiming students in QRDA and in ACDA. ⊖ denotes the values when *θ* = 0.1 and × denotes the values when *θ* = 0.3.

To compare the nonwastefulness of QRDA and ACDA, we measure the proportion of claiming students, i.e., students who claim an empty seat, in both mechanisms and show the difference in Fig 3, i.e., by plotting (|*S*_ACDA_| − |*S*_QRDA_|)/*n*, where *S*_ACDA_ (respectively, *S*_QRDA_) denotes the set of claiming students in ACDA (respectively, QRDA). This value is always positive for both *θ* = 0.1 and *θ* = 0.3, which means that on average more students claim empty seats in ACDA than in QRDA. When *θ* = 0.1, this number is 40% for *β* = 10 and increases to approximately 60% for *β* = 40 before plateauing. Similarly, this value is lower (less wasteful) for *θ* = 0.3 when *β* < 40 and higher (more wasteful) when *β* > 40. We conclude that QRDA is less wasteful than ACDA in a difference constraints setting. Similar to Fig 2, for both cases of *θ* = 0.1 and *θ* = 0.3, QRDA’s amelioration becomes greater as *β* increases.

**Fig 4:**
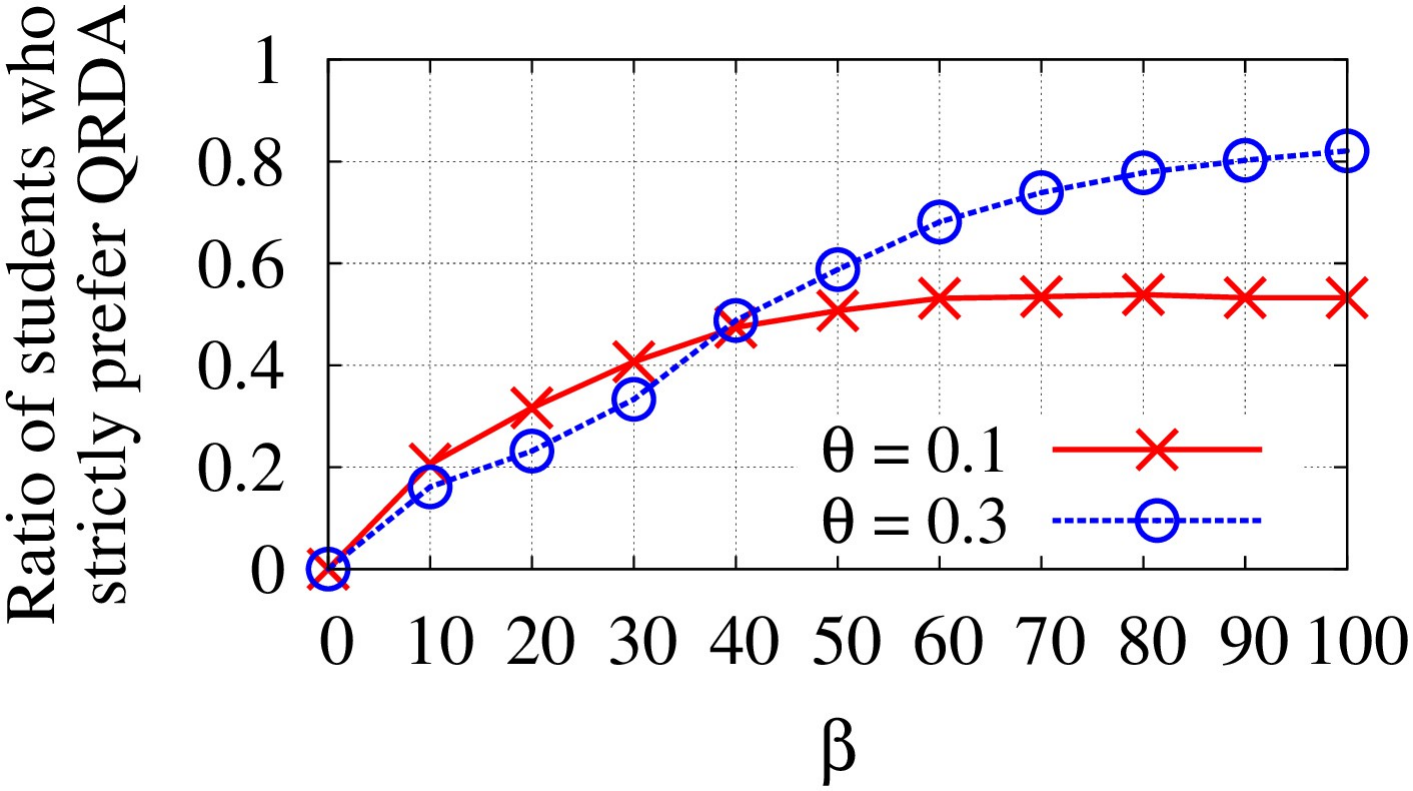
Comparisons between QRDA and ESDA under difference constraints: Ratio of students who strictly prefer QRDA to ESDA. ⊖ denotes the values when *θ* = 0.1 and × denotes the values when *θ* = 0.3.

We next compare QRDA and ESDA through similar simulations. In Fig 4, we plot the ratio of students who strictly prefer QRDA over ESDA depending on *β*. The trend closely resembles Fig 2. This is reasonable since, similar to ACDA, many students have to be assigned to less preferred schools to meet the minimum quotas in ESDA. The difference at the plateau is slightly smaller than that of Fig 2 when both *θ* = 0.1 and 0.3.

**Fig 5:**
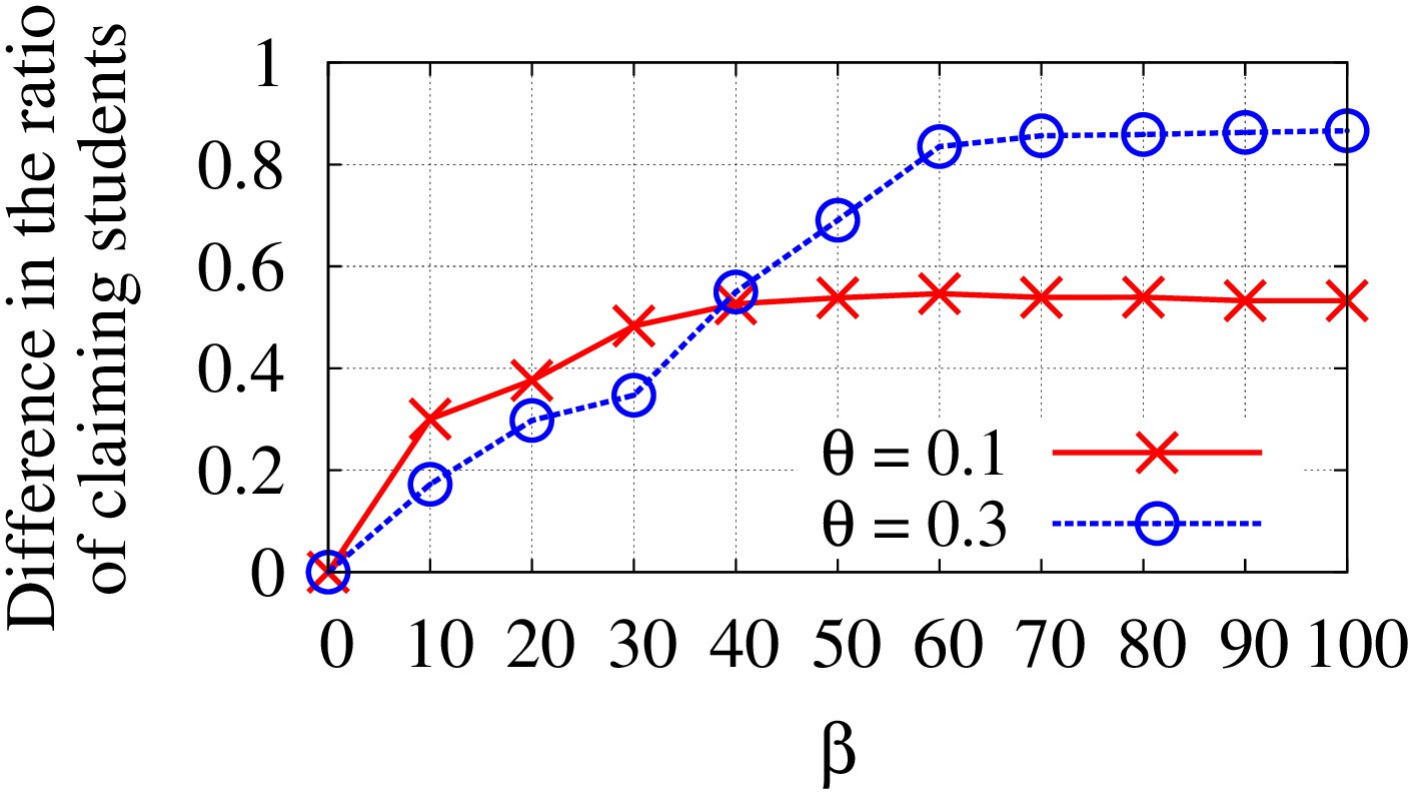
Comparisons between QRDA and ESDA under difference constraints: Difference between ratio of claiming students in QRDA and in ESDA. ⊖ denotes the values when *θ* = 0.1 and × denotes the values when *θ* = 0.3.

Fig 5 presents the difference in the ratio of the claiming students between QRDA and ESDA. The graph resembles Fig 3. Overall, we confirm that QRDA experimentally outperforms ACDA and ESDA under difference constraints.

Finally, we conduct computer simulations with *flexible uniform min/max quotas constraints* (Definition 17). Among the three variables, *p*, *q* and *d*, in these constraints, we choose to fix *q* = 60 and vary *p* and *d* since they have the greatest impact on feasibility. When varying *q*, the results were almost constant since maximum quotas only impact the most popular schools.

**Fig 6:**
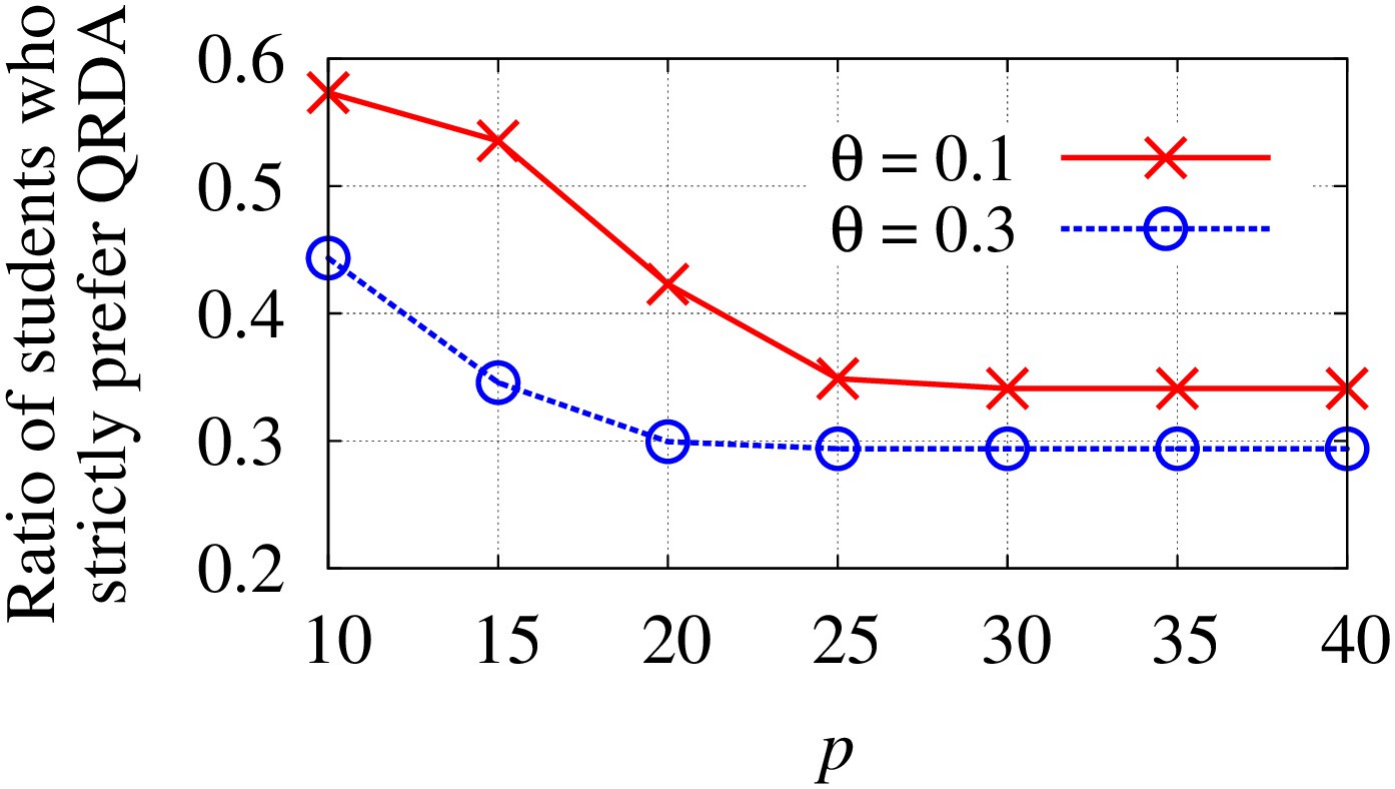
Comparisons between QRDA and ACDA under flexible uniform min/max quotas constraints: Ratio of students who strictly prefer QRDA to ACDA by varying *p*. ⊖ denotes the values when *θ* = 0.1 and × denotes the values when *θ* = 0.3.

**Fig 7:**
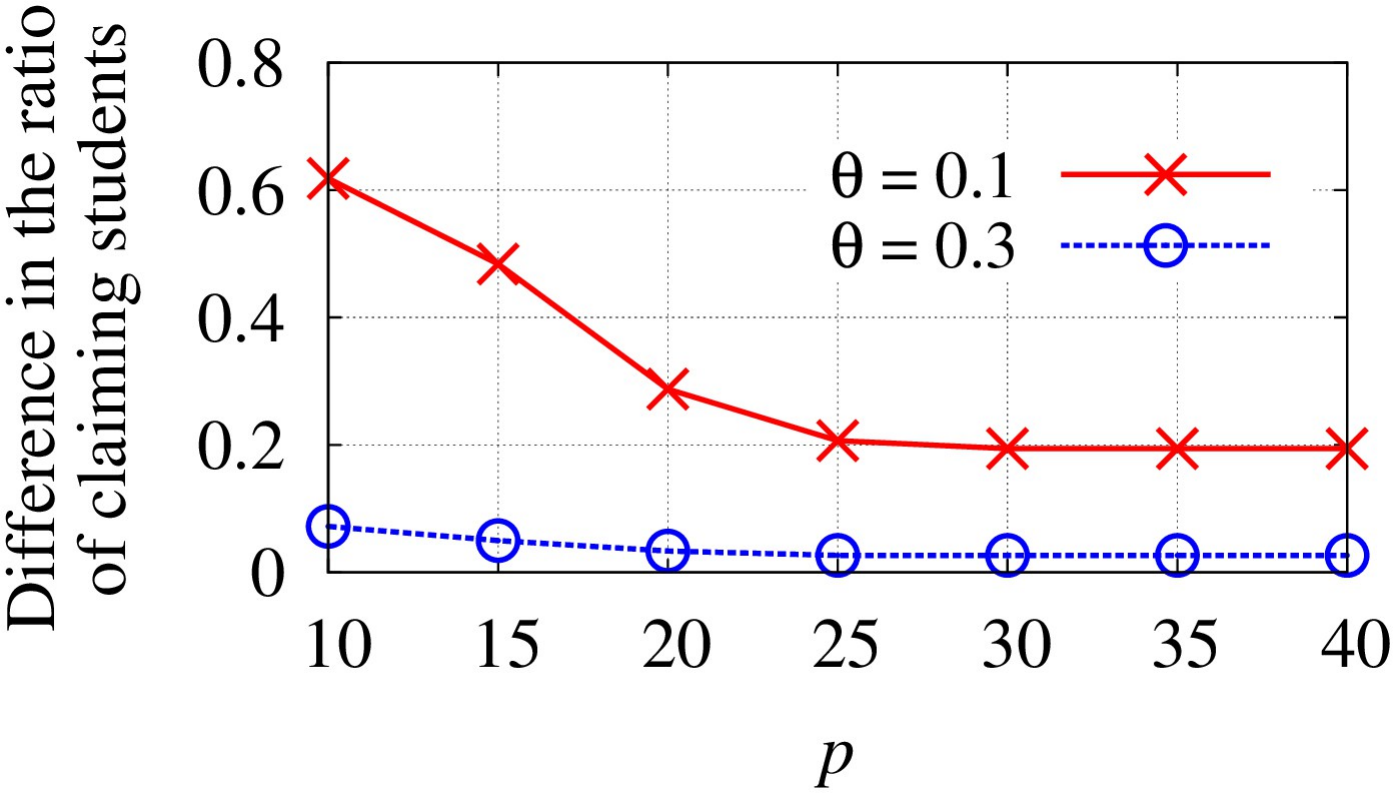
Comparisons between QRDA and ACDA under flexible uniform min/max quotas constraints: Difference between ratio of claiming students in QRDA and ACDA by varying *p*. ⊖ denotes the values when *θ* = 0.1 and × denotes the values when *θ* = 0.3.

Fig 6 shows the ratio of students who strictly prefer QRDA over ACDA, and Fig 7 shows the difference in the ratio of claiming students between QRDA and ACDA when minimum quota *p* is varied. We set *d* = 100. Both graphs gradually decrease as *p* increases until reaching a stabilized value. This result confirms that QRDA outperforms ACDA in this setting. It further shows that the improvement obtained by QRDA is limited when the minimum quotas are large. This is because a larger minimum quota can significantly shrink the constraint set, which leads to much less flexibility. Therefore, it limits the QRDA performance to a large extent.

**Fig 8:**
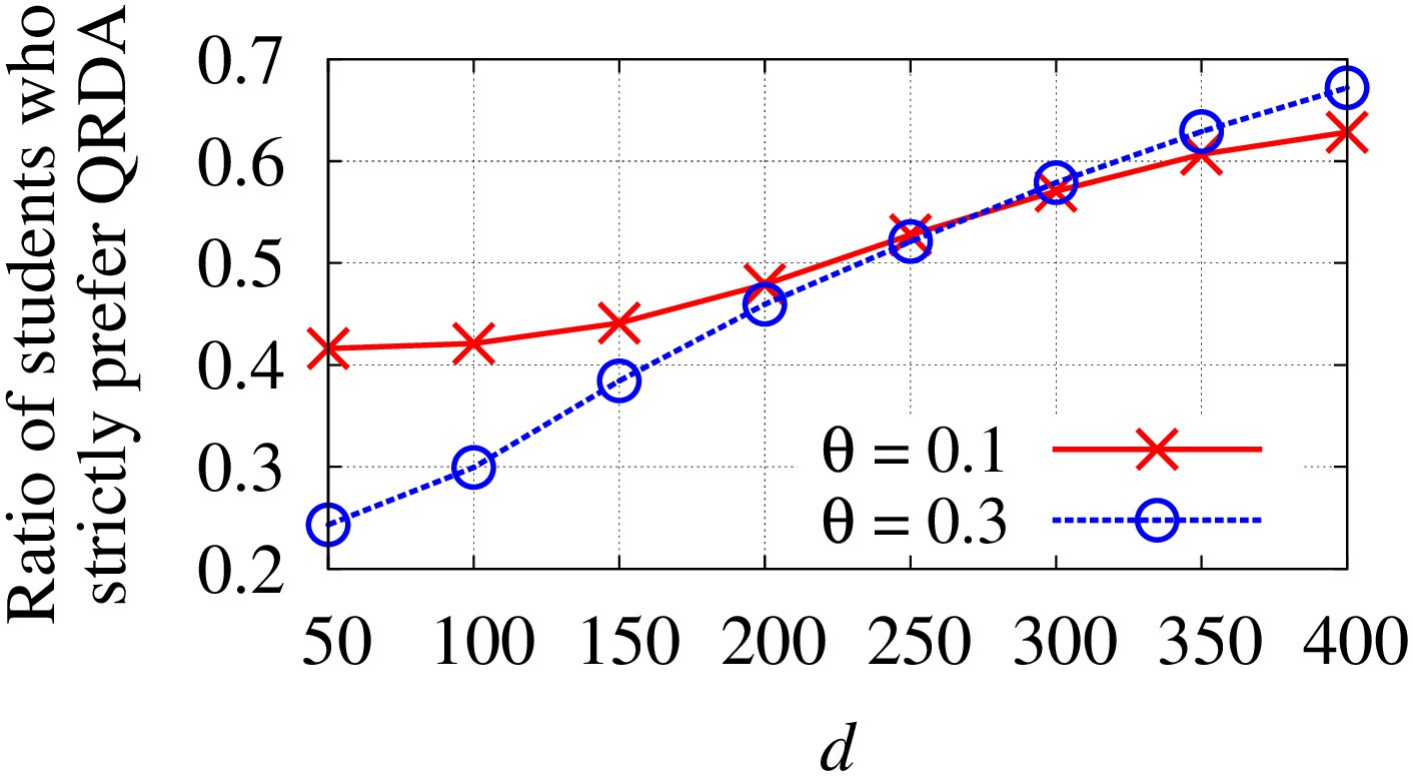
Comparisons between QRDA and ACDA under flexible uniform min/max quotas constraints: Ratio of students who strictly prefer QRDA to ACDA by varying *d*. ⊖ denotes the values when *θ* = 0.1 and × denotes the values when *θ* = 0.3.

**Fig 9:**
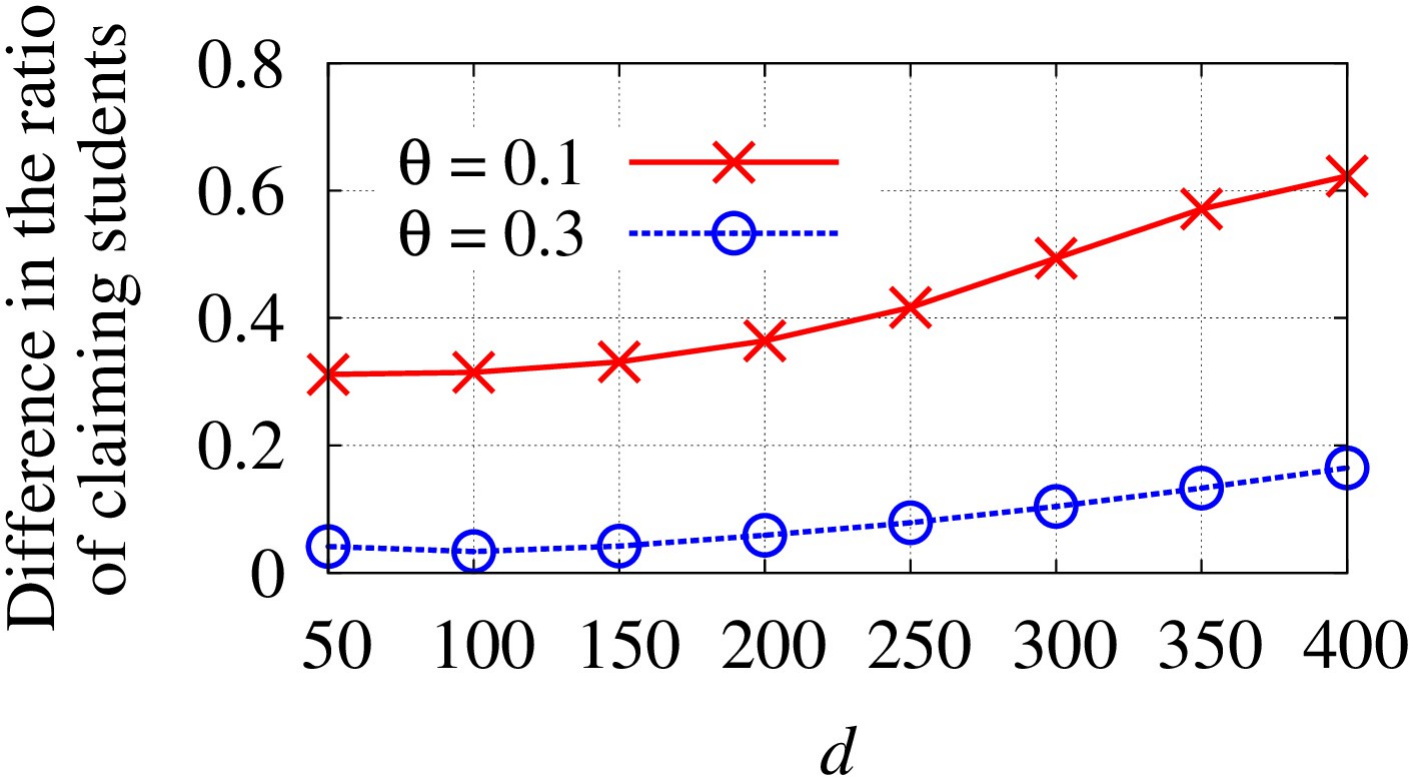
Comparisons between QRDA and ACDA under flexible uniform min/max quotas constraints: Difference between ratio of claiming students in QRDA and ACDA by varying *d*. ⊖ denotes the values when *θ* = 0.1 and × denotes the values when *θ* = 0.3.

We present similar simulations when varying distance parameter *d* in Figs 8 and 9. We set *p* = 20. Fig 8 shows that, as *d* increases, the ratio of students who strictly prefer QRDA over ACDA increases. Even with higher competition (*θ* = 0.3), QRDA significantly outperforms ACDA when *d* is large. In Fig 9, the trend is similar for *θ* = 0.1, but for the averages of *θ* = 0.3, the curve only increases slightly.

## 7 Conclusion and future work

This paper identified a groundbreaking class of distributional constraints, defined as a union of symmetric M-convex sets. Since M-convexity is not closed under union, a union of symmetric M-convex sets is not an M-convex set. We devised a fair and strategyproof mechanism named QRDA based on DA. Additionally, we demonstrated that QRDA inherits several significant axiomatic properties from DA such as weak group strategyproofness, weak Pareto optimality, or weak non-bossiness. Importantly, these theoretical results have been further verified in a generalized distributional constraints class: union of symmetric M-convex constraints with an offset. In terms of student welfare, we proved that QRDA theoretically outperforms ACDA. Moreover, through rigorous experimentation, we showed that QRDA outperforms both ACDA and ESDA in terms of student welfare and nonwastefulness.

We intend to clarify whether any class of constraints exists that is broader than the union of symmetric M-convex sets, where a non-trivial fair and strategyproof mechanism exists.
